# Oncolytic virotherapy enhances the efficacy of a cancer vaccine by modulating the tumor microenvironment

**DOI:** 10.1002/ijc.32325

**Published:** 2019-04-29

**Authors:** Iris Koske, Annika Rössler, Lisa Pipperger, Monika Petersson, Isabel Barnstorf, Janine Kimpel, Christoph H. Tripp, Patrizia Stoitzner, Zoltán Bánki, Dorothee von Laer

**Affiliations:** ^1^ Division of Virology Medical University of Innsbruck Innsbruck Austria; ^2^ ViraTherapeutics GmbH Innsbruck Austria; ^3^ Department of Dermatology, Venereology & Allergology Medical University of Innsbruck Innsbruck Austria

**Keywords:** melanoma, oncolytic virotherapy, DC‐based immunotherapy, tumor microenvironment, CD8 T cells

## Abstract

The efficacy of cancer vaccines has been limited by the immunosuppressive tumor microenvironment, which can be alleviated by immune checkpoint inhibitor (ICI) therapy. Here, we tested if oncolytic viruses (OVs), similar to ICI, can also synergize with cancer vaccines by modulating the tumor microenvironment. VSV‐GP, a chimeric vesicular stomatitis virus (VSV) pseudotyped with the glycoprotein (GP) of the lymphocytic choriomeningitis virus, is a promising new OV candidate. Here, we show that in mouse B16‐OVA melanoma, combination treatment of VSV‐GP with an ovalbumin (OVA) peptide‐loaded dendritic cell (DC) vaccine (DCVacc) significantly enhanced survival over the single agent therapies, although both DCVacc and DCVacc/VSV‐GP treatments induced comparable levels of OVA‐specific CD8 T cell responses. Virus replication was minimal so that direct viral oncolysis in B16‐OVA did not contribute to this synergism. The strong therapeutic effect of the DCVacc/VSV‐GP combination treatment was associated with high numbers of tumor‐infiltrating, highly activated T cells and the relative reduction of regulatory T cells in treated and contra‐lateral nontreated tumors. Accordingly, depletion of CD8 T cells but not natural killer cells abrogated the therapeutic effect of DCVacc/VSV‐GP supporting the crucial role of CD8 T cells. In addition, a drastic increase in several proinflammatory cytokines was observed in VSV‐GP‐treated tumors. Taken together, OVs, similar to ICI, have the potential to markedly increase the efficacy of cancer vaccines by alleviating local immune suppression in the tumor microenvironment.

## Abstract 2

**What's new?**

Cancer vaccine efficacy has been limited by the immunosuppressive tumor microenvironment. By inducing cancer cell death with the release of tumor‐related antigens, oncolytic viruses may have an adjuvant effect. Here, the authors show that a combination of the oncolytic rhabdovirus VSV‐GP and a dendritic cell vaccine is highly effective in the treatment of mouse melanoma, most likely because VSV‐GP reprograms the tumor microenvironment to enhance the effectivity of the vaccine‐induced immune response. Oncolytic viruses have the potential to dramatically increase the efficacy of cancer vaccines by alleviating local immune suppression in the tumor microenvironment.

**Abbreviations**
CTL: cytotoxic T lymphocyte
DC: dendritic cells
DCVacc: CpG‐matured, OVA‐loaded bone marrow‐derived DC
GFP: green fluorescence protein
ICI: immune checkpoint inhibitor
OV: oncolytic virus
OVA: ovalbumin
TIL: tumor infiltrating lymphocytes
VSV: vesicular stomatitis virus
VSV‐GP: a chimeric VSV pseudotyped with the glycoprotein of the lymphocytic choriomeningitis virus

## Introduction

Cancer immunotherapies aim to stimulate a potent antitumor immune response and to overcome the immunosuppressive tumor microenvironment.^1^ Due to their crucial role in priming T cell responses, dendritic cells (DC) have been extensively studied as a tool for cancer vaccination.^2^ The safety and ability to induce antitumor responses have clearly been established for DC vaccination and patients with long‐term benefit have been reported.^3^ Still, DC vaccines have not yet fulfilled their promise and still face several challenges^4^ as only a small number of injected DCs migrate to draining lymph nodes^5^, ^6^ and their optimal activation is critical.^7^ Recent evidence from DC‐based vaccinations against melanoma suggests that efficacy depends on several factors such as the mode of antigen loading, the maturation‐state of the DCs, the site of injection and the vaccination schedule.^8^, ^9^, ^10^, ^11^, ^12^ Finally, local immunosuppression in the tumor microenvironment is expected to reduce the therapeutic effect of DC vaccinations in the majority of cancer types.^13^

Oncolytic viruses (OV) also represent promising therapeutic options for the treatment of cancer as they selectively replicate in cancer cells and kill them. VSV‐GP, the vesicular stomatitis virus (VSV) pseudotyped with the lymphocytic choriomeningitis virus (LCMV) glycoprotein (GP), shows oncolytic capacity comparable to VSV in different tumor entities.^14^ Pseudotyping of VSV with LCMV GP completely abrogates the VSV's neurotoxicity and reduces the induction of virus‐neutralizing antibodies.^15^, ^16^

In addition to direct oncolysis, OVs can act as a cancer vaccine by inducing immunogenic cell death with the release of tumor‐related antigens, which then induce an antitumoral immune response.^17^ Here, the OV infection has an adjuvant effect as it activates danger signals causing local inflammation by stimulation of innate immune cells, and pronounced Th1 polarization of the adaptive immune response.^18^, ^19^, ^20^ Thus, we propose that VSV‐GP oncolytic virotherapy acts as a natural adjuvant for DC‐based tumor vaccination and helps to overcome the local immune suppression in tumor tissues. To test this prediction, we used the mouse melanoma B16, in which VSV‐GP replicates inefficiently and the pure lytic activity of the virus does not contribute significantly to the therapeutic effect.^21^ Indeed, in B16 melanomas, VSV‐GP and a DC vaccine act synergistically. The data support a mode of action where the DC vaccine induces tumor‐specific T cells, while VSV‐GP alters the tumor microenvironment to overcome local immune suppression.

## Materials and Methods

### Cell lines

B16‐OVA melanoma (kindly provided by D.M. Brown^22^) was cultured in DMEM (Lonza, Switzerland) with 10% FCS (PAA Laboratories, Austria), 2% glutamine (Gibco, Waltham, MA), 100 U/ml penicillin (Gibco), 0.1 mg/ml streptomycin (Gibco) and 0.5 μg/ml geneticin (G418) sulfate (Santa Cruz Biotechnology, Dallas, TX). L929 cells (DSMZ GmbH, Germany) were cultured treated with either (i) PBS 10% FCS, 2% glutamine, 100 U/ml penicillin and 0.1 mg/ml streptomycin. BHK‐21 cells (ATCC, Manassas, VA) were cultured in GMEM (Gibco) with 10% FCS, 5% tryptose phosphate broth (Gibco), 100 U/ml penicillin and 0.1 mg/ml streptomycin.

### Mice

Six to eight weeks old female C57BL/6J mice (Harlan Laboratories, Germany and Janvier, France) were housed under specific pathogen‐free conditions. Animal experiments were approved by the ethics committees of the Medical University of Innsbruck and the Austrian Federal Ministry of Science and Research (BMWF‐66.011/0092‐WF/V/3b/2016; BMWF‐66.011/0119‐II/3b/2012).

### Viruses

Recombinant VSV‐GP and the green fluorescence protein (GFP)‐encoding VSV‐GP‐GFP were described previously^15^ and generated on L929 or BHK‐21 cells. Virus was titrated on BHK‐21 using plaque assay.

### Generation of bone marrow‐derived DCs and DC vaccination

Bone marrow‐derived DCs (bmDC) were generated in the presence of recombinant mouse GM‐CSF (4 ng/ml, BD Bioscience, San Jose, CA) and IL‐4 (4 ng/ml, BD Bioscience) as described previously.^23^ At Day 7, DCs were matured by overnight incubation with 2 μg/ml CpG ODN 1826 (InvivoGen, San Diego, CA). On Day 8, CpG‐matured bmDCs were pulsed with 10 μM chicken ovalbumin (OVA)‐derived SIINFEKL peptide (InvivoGen) for 3 hr, subsequently, cells were washed in PBS (Lonza, Switzerland) and a total of 2 × 10^5^ cells in 50 μl were injected partly intratumorally and peritumorally (i.t./p.t.) or 2 × 10^5^ cells in 100 μl i.v. into the mice and referred as DCVacc or sysDCVacc, respectively.

### Tumor challenge and treatments

5 × 10^5^ B16‐OVA cells were s.c. injected in the right flank of C57BL/6J mice. Mice were treated i.t./p.t. on Day 11, 18 and 25 posttransplantation (at a tumor size of around 0.025 cm^3^) either with 50 μl PBS, 6 × 10^7^ PFU VSV‐GP (VSV‐GP), DCVacc or a combination of DCVacc and 6 × 10^7^ PFU VSV‐GP (DCVacc/VSV‐GP). Alternatively, DCVacc was applied systemically either alone by administering sysDCVacc or i.v. application of DCVacc was combined with 6 × 10^7^ PFU VSV‐GP injected i.t./p.t. (sysDCVacc/VSV‐GP). Cured DCVacc/VSV‐GP treated animals were rechallenged with either 5 × 10^5^ B16 or B16‐OVA cells. In experiments using bilateral tumors, 5 × 10^5^ B16‐OVA cells were s.c. injected into the right flank of C57BL/6 mice. Three days later 5 × 10^5^ B16‐OVA were s.c. injected into the left flank of the mice. On Day 10, posttransplantation right flank tumors were treated either with 50 μl PBS, VSV‐GP, DCVacc or DCVacc/VSV‐GP. Mice were analyzed 7 days posttreatment. Tumor size was determined by measuring the length (*L*) and width (*W*) using a caliper every 1–2 days. The tumor volume (cm^3^) was calculated with the formula 0.4 × *L* × *W* ^2^. For histological analysis, mice were treated i.t. with either 30 μl PBS (control) or VSV‐GP‐GFP (5 × 10^8^ TCID_50_) at a tumor size between 0.05 and 0.1 cm^3^ and analyzed 1 day or 6 days posttreatment.

### FACS analysis

The antibodies used are listed in Supporting Information Table S1. For tetramer staining, 30 μl blood collected from the tail vein was stained with SIINFEKL‐APC tetramer for 20 min at 37°C, followed by surface antibody staining for 30 min at 4°C. Erythrocytes were lysed with ACK buffer and cells were washed and fixed with PBS containing 1% formaldehyde (Carl Roth, Germany). Spleen and tumors were weighed and processed for flow cytometry analysis and *in vitro* cytokine production. Spleens were pressed through 100 μm cell strainers (BD Biosciences, San Jose, CA), prior to lysis of erythrocytes with ACK buffer. Cell suspensions were filtered through 70 μm cell strainer. Tumors were minced with scissors and digested in RPMI with 0.8 mg/ml Dispase II, 0.2 mg/ml collagenase P, 0.1 mg/ml DNase I (all from Roche, Switzerland) for 30 min at 37°C. Isolated cells from B16‐OVA tumors were filtered through 70 μm cell strainer and purified on Ficoll gradient (Cedarlane Laboratories, Burlington, ON, Canada). Splenocytes or cells from B16‐OVA tumors (1 × 10^6^) were stained with monoclonal antibodies for 30 min at 4°C. To detect FoxP3 positive regulatory T cells, mouse regulatory T cell staining kit (eBioscience, San Diego, CA) was used according to manufactory instruction. For intracellular cytokine stainings, 2 × 10^6^ splenocytes or cells from B16‐OVA tumors were stimulated with 5 μg/ml OVA (SIINFEKL) or VSV N (RGYVYQGL) peptide (Genscript, Piscataway, NJ) in RPMI with 10% FCS and 2 μl/ml GolgiPlug (BD Bioscience) for 6 hr at 37°C. As negative control, cells were cultured without peptides. Intracellular cytokine staining was performed using Cytofix/Cytoperm kit (BD Bioscience) according to the manufacture's protocol. Samples were measured using a FACSCanto II cytometer (BD Bioscience) and data were analyzed using FACSDiva (BD Bioscience) or FlowJo (Tree Star, Ashland, OR) software.

### Measuring cytokines in tumor lysates

Tumors were collected and digested in Invitrogen™ ProcartaPlex™ cell lysis buffer (ThermoFischer Scientific, Austria) using SpeedMill Plus homogenizator (AnalytikJena, Germany). Tumor lysates were stored at −80°C until use. Cytokines were determined using LEGENDPlex™ mouse inflammation panel (BioLegend, Germany) according to the manufacture's protocol. IL‐28 was measured by IL‐28 ELISA kit from PBL Assay Science (Piscataway, NJ) according to the manufacture's protocol.

### Depletion of natural killer and CD8 T cells *in vivo*

Natural killer (NK) cells and CD8 T cells were depleted using anti‐NK1.1 (clone PK136, 250 μg/treatment; BioXCell, West Lebanon, NH) and anti‐CD8 (clone YTS 169.4, 100 μg/treatment; BioXCell) antibodies, respectively, by i.p. injection in 100 μl PBS on Day 8, 10, 14, 17 and 21 posttumor transplantation. Isotype controls IgG2a (clone C1.18.4, 100 μg/treatment; BioXCell) and IgG2b (100 μg/treatment; BioXCell) served as negative controls for anti‐NK1.1 and anti‐CD8, respectively. Treatment with DCVacc/VSV‐GP was started 3 days after the first injection of the depleting antibodies and repeated twice in 7 days interval.

### Statistics

Statistical analyses were performed using GraphPad Prism software (GraphPad Software, Inc., Chicago, IL). For comparison of multiple groups, data were analyzed using one‐way ANOVA with Tukey's multiple comparison tests. Survival curves were represented as Kaplan–Meier survival plots and analyzed using the Mantel–Cox test. Two‐sided *p* < 0.05 was considered statistically significant (*p ≤ 0.05,**p ≤ 0.01,***p ≤ 0.001,****p ≤ 0.0001).

## Results

### Combination of oncolytic VSV‐GP with DC vaccination is highly effective in B16‐OVA melanoma model

**Figure 1:**
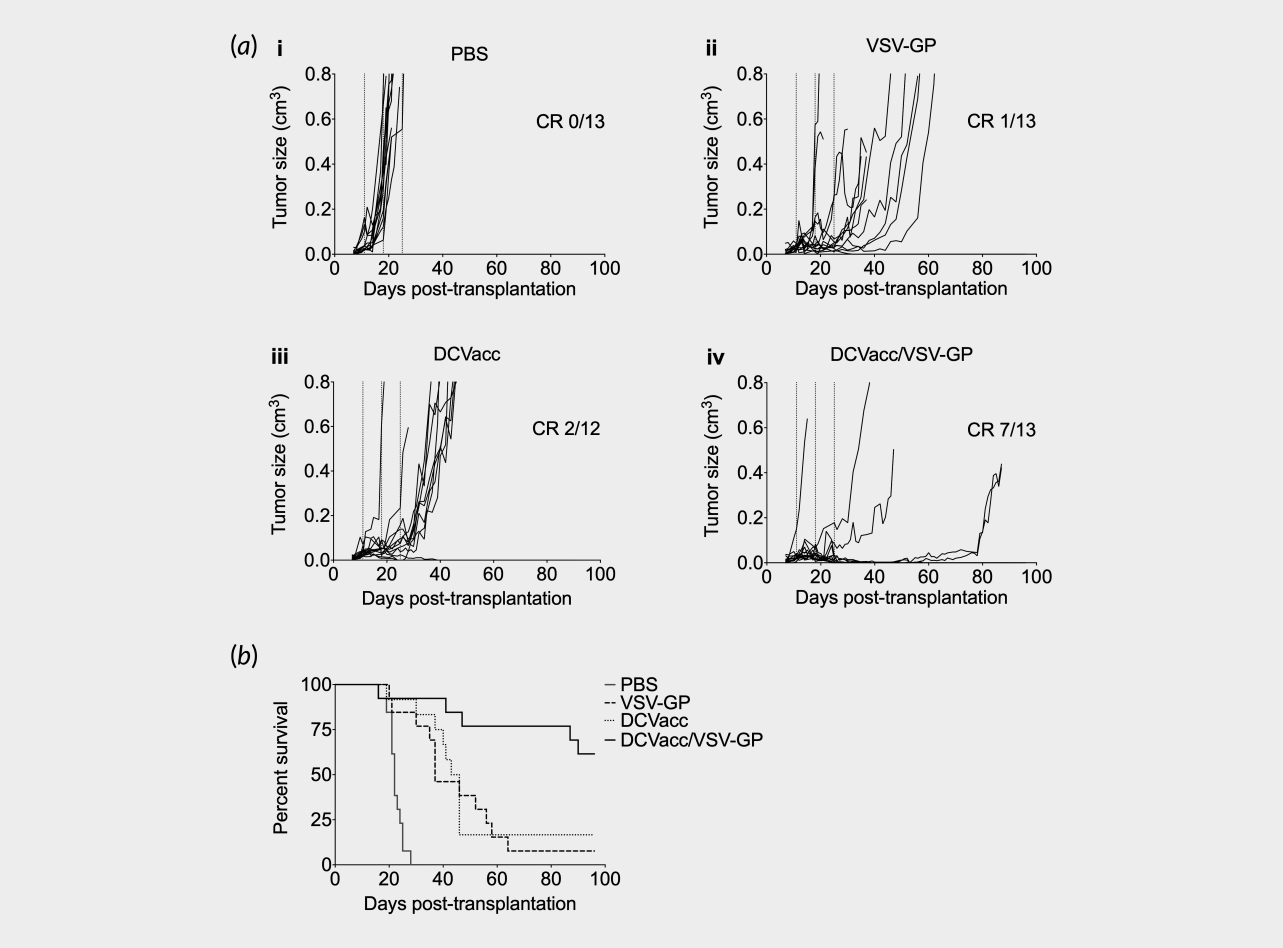
DCVacc/VSV‐GP combination therapy showed significant survival benefit compared to single treatments. (*a*) Tumor growth curves of s.c. B16‐OVA melanoma in C57BL/6 mice treated i.t./p.t. on Days 11, 18 and 25 posttransplantation with PBS, VSV‐GP (6 × 10^7^ PFU), DCVacc (2 × 10^5^ SIINFEKL‐loaded CpG‐matured bmDCs) or DCVacc/VSV‐GP. Number of mice with a complete remission (CR) to the treatment are indicated in the Figures. (*b*) Overall survival. Data were analyzed by Mantel–Cox test. PBS *vs*. VSV‐GP = *p* < 0.0001; PBS *vs*. DCVacc = *p* < 0.0001; PBS *vs*. DCVacc/VSV‐GP = *p* < 0.0001. DCVacc/VSV‐GP *vs*. VSV‐GP = *p* < 0.01 and DCVacc/VSV‐GP *vs*. DCVacc = *p* < 0.01. Data represent results from two independent experiments with a total of *n* = 12–13 mice per group.

The combination of oncolytic VSV‐GP virotherapy with DC vaccination was investigated in the syngeneic subcutaneous B16‐OVA mouse model. B16‐OVA cells were implanted s.c. into the right flank skin of C57BL/6 mice and animals were treated with either (*i*) PBS, (*ii*) VSV‐GP (VSV‐GP), (*iii*) CpG‐matured OVA‐peptide loaded dendritic cells (DCVacc) or (*iv*) a mixture of DCVacc and VSV‐GP (DCVacc/VSV‐GP) by intratumoral and peritumoral (i.t./p.t.) injection. All treatments were repeated twice in 7‐day intervals. Both, DCVacc and VSV‐GP single treatments resulted in a comparable delay of tumor growth and significantly prolonged survival compared to PBS‐treated control mice (Figs. 1a and 1b). In the DCVacc/VSV‐GP combination group, tumor growth was further delayed and survival improved considerably compared to the single treatments (Figs. 1a and 1b). Seven of 13 treated animals showed long‐term complete remission. To see if these cured animals had established an effective antitumoral immune response, we injected these animals 90 days after implantation of the primary tumors with either B16‐OVA cells or with the parental B16 cells. In the rechallenge experiment, all mice cured of B16‐OVA tumors by DCVacc/VSV‐GP combination treatment were immune to a secondary challenge with B16‐OVA (Supporting Information Fig. S1). Secondary challenge of cured mice with B16 parental cells resulted in a delay of tumor growth and an improved survival compared to the naive control group, nevertheless none of the four rechallenged mice were completely immune (Supporting Information Fig. S1). These data indicated that the immune response in the DCVacc/VSV‐GP treated mice was primarily focused on the OVA‐antigen but that some antigen spread was induced and responses were also directed against nontargeted B16‐associated tumor antigens.

The systemic application of DCVacc (sysDCVacc) through intravenous injection also resulted in a delay in tumor progression as well as in a significantly improved survival, similarly to that seen when treatment was performed i.t./p.t. (Supporting Information Figs. S2A and S2B). Again, the combination of VSV‐GP i.t./p.t. treatment with sysDCVacc showed better tumor control and significantly improved survival over sysDCVacc treatment alone (Supporting Information Figs. S2A and S2B). However, sysDCVacc combined with an i.t./p.t. VSV‐GP treatment seemed to be less potent than i.t./p.t. DCVacc/VSVGP treatment, but the differences did not reach significance in survival. Therefore, the local coapplication of DCVacc and VSV‐GP does not seem to be essential for therapeutic activity.

### Transient VSV‐GP‐GFP replication in the B16‐OVA tumors

We then studied virus spread and virus‐induced cell death of i.t. VSV‐GP‐GFP treatment in the B16‐OVA syngeneic melanoma model on Day 1 and Day 6 posttreatment by immunohistology (as described in Supporting Information Material and Methods). At Day 1, after VSV‐GP administration, approximately 5–10% of the cells in the tumor were GFP positive and located in patches of apoptotic cells as detected by an anticaspase‐3 antibody. Six days posttreatment GFP positive cells could barely be found, but strong caspase‐3 signals were still detectable, indicating that apoptosis was still ongoing (Supporting Information Fig. S3). These data clearly show that VSV‐GP induced tumor cell death, but that viral spread was limited within the tumor.

### DCVacc and DCVacc/VSV‐GP induce comparable levels of OVA‐specific CD8 T cell responses

**Figure 2:**
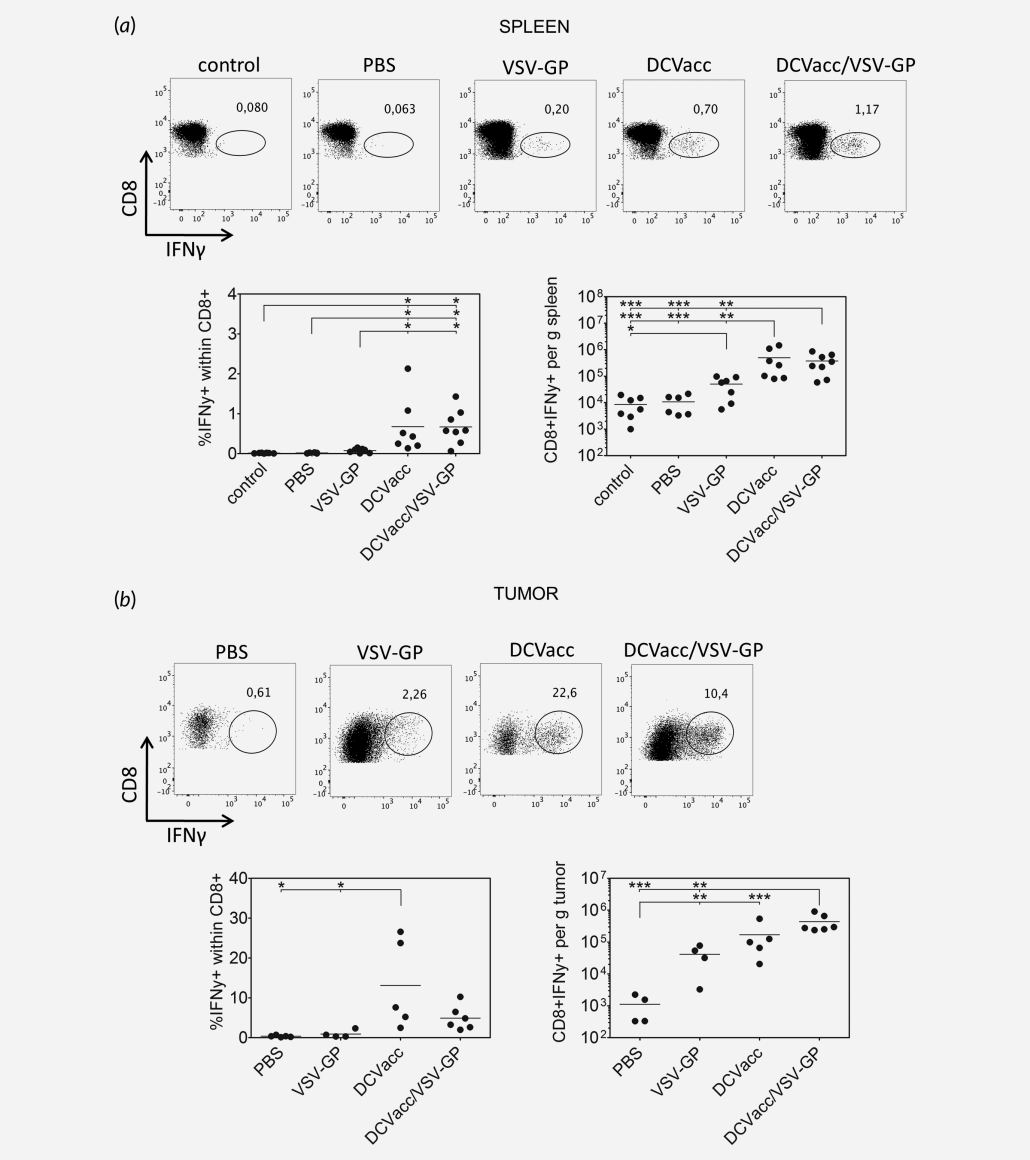
Comparable levels of OVA‐specific IFNγ producing CD8 T cells upon DCVacc and DCVacc/VSV‐GP therapies. B16‐OVA melanoma in C57BL/6 mice were treated i.t./p.t. on Days 11 and 18 posttransplantation with PBS (control), VSV‐GP (6 × 10^7^ PFU), DCVacc (2 × 10^5^ OVA‐loaded CpG‐matured bmDCs) or DCVacc/VSV‐GP. Seven days after the second treatment spleen (*a*) and cells from B16‐OVA tumors (*b*) were isolated. Cells were stimulated *in vitro* with OVA peptide and the production of IFNγ was measured by FACS. FACS dot plots depicting CD8 positive cells (*y*‐axis) and intracellular IFNγ (*x*‐axis) within the CD45+ CD3+ cell population show representative data from the different treatment groups. Data represent cumulative results of two independent experiments. Data were analyzed by ANOVA followed by Tukey's multiple comparisons test (*p ≤ 0.05,**p ≤ 0.01,***p < 0.001).

The relatively low percentage of VSV‐GP infected cells in the tumor suggested that direct oncolysis did not contribute significantly to the therapeutic effect of VSV‐GP alone or in combination with DCVacc. The rechallenge experiments rather suggest that mice in the DCVacc/VSV‐GP treated group had mounted a potent immune response primarily against OVA. We thus measured OVA‐specific responses 7 days after the second VSV‐GP or DCVacc single and combination treatments in spleen and tumor tissue by measuring IFNγ‐producing CD8 T cells after *in vitro* OVA‐peptide stimulation. OVA‐specific IFNγ‐producing CD8 T cells in spleen and tumor, as well as OVA‐tetramer positive CD8 T cells in the blood, were clearly detectable in the VSV‐GP group but only in about 30% of the mice (Figs. 2 *a* and 2 *b*, Supporting Information Fig. S4). This suggests that antigens released by the oncolytic activity of VSV‐GP can support antigen presentation and the induction of tumor‐specific CTLs, although relatively inefficiently. In contrast, DCVacc and DCVacc/VSV‐GP therapy induced a clear OVA‐specific CD8 T cell response in the spleen of all animals (Fig. 2 *a*) and responses in both groups were comparable. Similarly, there was no significant difference in the total number of OVA‐specific CD8 T cells found in tumor tissues between DCVacc and DCVacc/VSV‐GP treated mice (Fig. 2 *b*). We even found a significantly lower percentage of OVA‐specific cells within the blood CD8 T cell compartment 7 days after the second DCVacc/VSV‐GP treatment when compared to the DCVacc group (Supporting Information Fig. S4). These data indicate that the improved efficacy of DCVacc/VSV‐GP relative to the single treatments was not due to a higher number of OVA‐specific CD8 T cells.

### Combination of DCVacc with oncolytic VSV‐GP increases the number of tumor‐infiltrating lymphocytes

**Figure 3:**
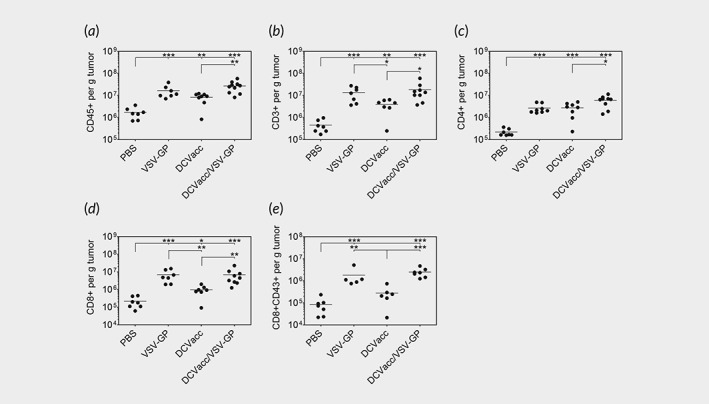
DCVacc/VSV‐GP combination treatment increased the numbers of tumor infiltrating lymphocytes (TIL). B16‐OVA melanoma in C57BL/6 mice were treated i.t./p.t. on Days 11 and 18 posttransplantation with PBS (control), VSV‐GP (6 × 10^7^ PFU), DCVacc (2 × 10^5^ OVA‐loaded CpG‐matured bmDCs) or DCVacc/VSV‐GP. Seven days after the second treatment cells from B16‐OVA tumors were isolated and TILs were analyzed by FACS. Total cell numbers per g tumor were calculated for CD45 positive immune cells (*a*), CD3 T cells (*b*), CD4 T cells (*c*), CD8 T cells (*d*) and activated CD43 positive CD8 T cells (*e*). Data represent results from two independent experiments. Data were analyzed by ANOVA followed by Tukey's multiple comparisons test (*p ≤ 0.05,**p ≤ 0.01,***p < 0.001).

The presence of tumor infiltrating lymphocytes (TIL) has been demonstrated as a positive prognostic marker in several cancer types.^24^ As the number of OVA‐specific CD8 T cells did not explain improved therapeutic efficacy of the DCVacc/VSV‐GP over the single DCVacc treatment, we next determined the number of TILs in tumor tissues 7 days after the second DCVacc and VSV‐GP single and combination treatments. The single VSV‐GP and DCVacc, as well as the DCVacc/VSV‐GP combination therapy significantly elevated the absolute number of CD45+ infiltrating immune cells per gram tumor compared to the PBS control group and a significant increase of CD45+ immune cells upon DCVacc/VSV‐GP treatment compared to DCVacc alone was seen (Fig. 3 *a*). Moreover, we found that all treatment regimens resulted in significantly higher CD3 T cell numbers in the tumor compared to PBS and that VSV‐GP and DCVacc/VSV‐GP treatments resulted in a significantly elevated number of CD3 T cells compared to the DCVacc group (Fig. 3 *b*). Similar effects were also seen for the CD8 T cell subset, whereas DCVacc/VSV‐GP combination treatment showed the strongest effect on the tumor‐infiltrating CD4 T cells. When compared to DCVacc alone or PBS control groups, VSV‐GP alone or in combination with DCVacc also significantly increased the absolute number of intratumoral CD8 T cells (Fig. 3 *d*) as well as the number of activated CD8 T cells determined by the expression of CD43 (Fig. 3 *e*). Taken together, inclusion of VSV‐GP into the treatment regimen led to an increase in the number of activated CD8 T cells in the tumor relative to the PBS and DCVacc groups, whereby the number of OVA‐specific cells was not higher in the DCVacc/VSV‐GP than in the DCVacc group.

### VSV‐GP treatments induce strong virus‐specific CD8 T cell responses

**Figure 4:**
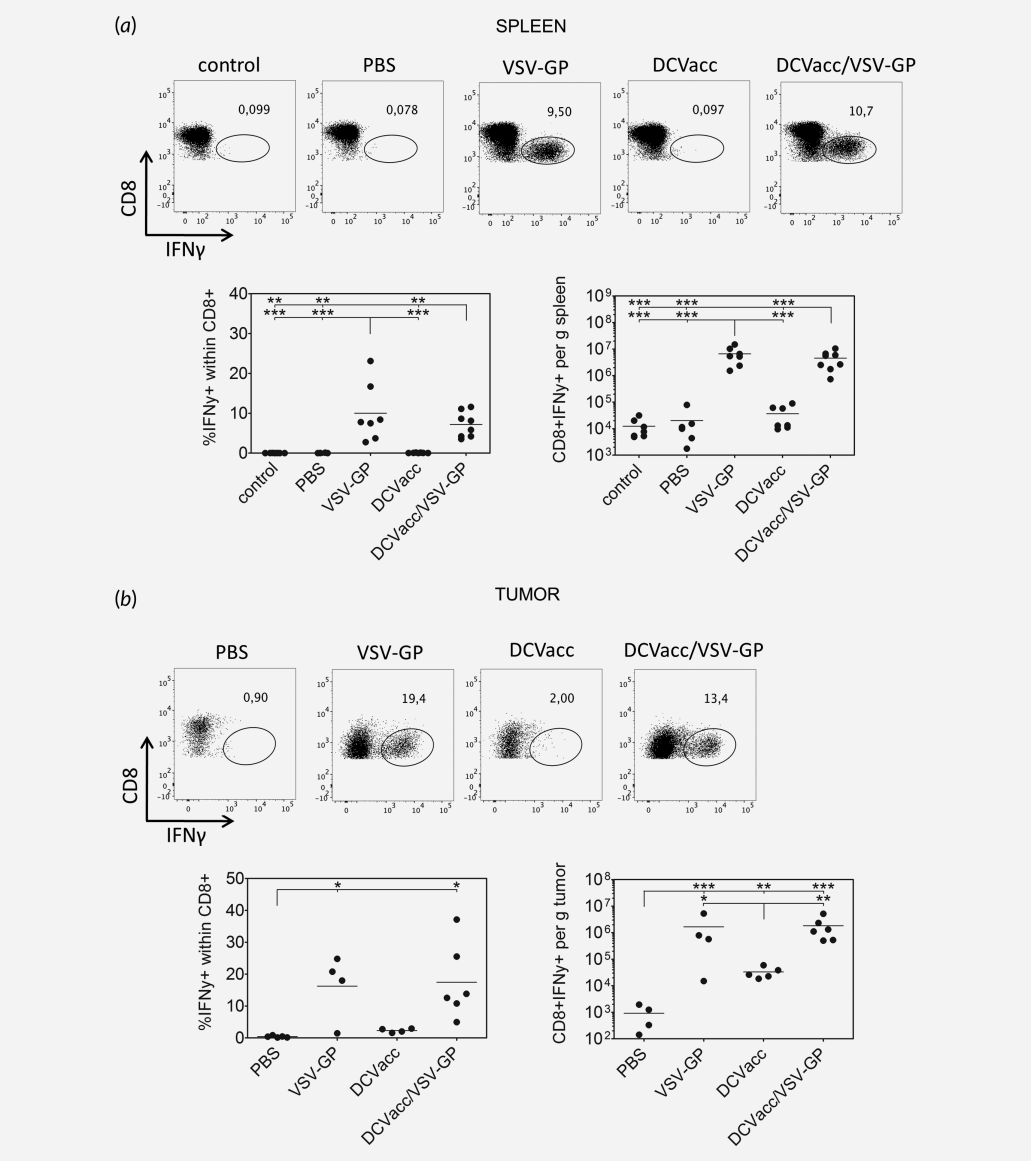
VSV N peptide‐specific IFNγ producing CD8 T cells in the spleen (*a*) and the tumor tissue (*b*). B16‐OVA melanoma in C57BL/6 mice were treated i.t./p.t. on Days 11 and 18 posttransplantation with PBS (control), VSV‐GP (6 × 10^7^ PFU), DCVacc (2 × 10^5^ OVA‐loaded CpG‐matured bmDCs) or DCVacc/VSV‐GP. Seven days after the second treatment spleen and cells from B16‐OVA tumors were isolated. To study VSV‐GP specific CD8 T cell responses, isolated spleen and cells from B16‐OVA tumors were stimulated *in vitro* with the VSV‐N‐derived immunodominant peptide RGYVYQGL and the production of IFNγ was measured by FACS. FACS dot plots depicting CD8 positive cells (*y*‐axis) and intracellular IFNγ (*x*‐axis) show representative data from the different treatment groups. Data represent results from two independent experiments. Data were analyzed by ANOVA followed by Tukey's multiple comparisons test (*p ≤ 0.05,**p ≤ 0.01,***p < 0.001).

We then studied if the strong activation of CD8 T cells in the groups that were treated with VSV‐GP reflected a potent anti‐viral CD8 T cell response. Thus, we analyzed the induction of VSV‐GP‐specific CD8 T cells by measuring IFNγ‐producing CD8 T cells in the spleen and tumors after *in vitro* restimulation with the VSV‐GP N‐protein‐derived immunodominant peptide RGYVYQGL. Indeed, inclusion of VSV‐GP in the regimen induced a high percentage of N‐peptide specific T cells in the spleen and tumor (Figs. 4 *a* and 4*b*). In addition, highly activated CD8 T cells could be seen in blood after VSV‐GP treatment (Supporting Information Fig. S5). These results support a strong induction of virus‐specific T‐cell responses most likely with a Th1 bias. Surprisingly, we also detected a few IFNγ‐producing CD8 T cells upon stimulation with the N‐peptide in tumor tissues of DCVacc treated mice. We cannot exclude that virus‐specific CD8 cells are somehow generated and recruited to the tumors after DCVacc, but more likely this phenomenon is caused by residual stimulation of OVA‐specific CD8 T cells with OVA protein from tumor cells in the cell preparation.

### Combination of DCVacc with oncolytic VSV‐GP elevates both CD4 T cell/Treg and CD8 T cell/Treg ratios within the tumor

**Figure 5:**
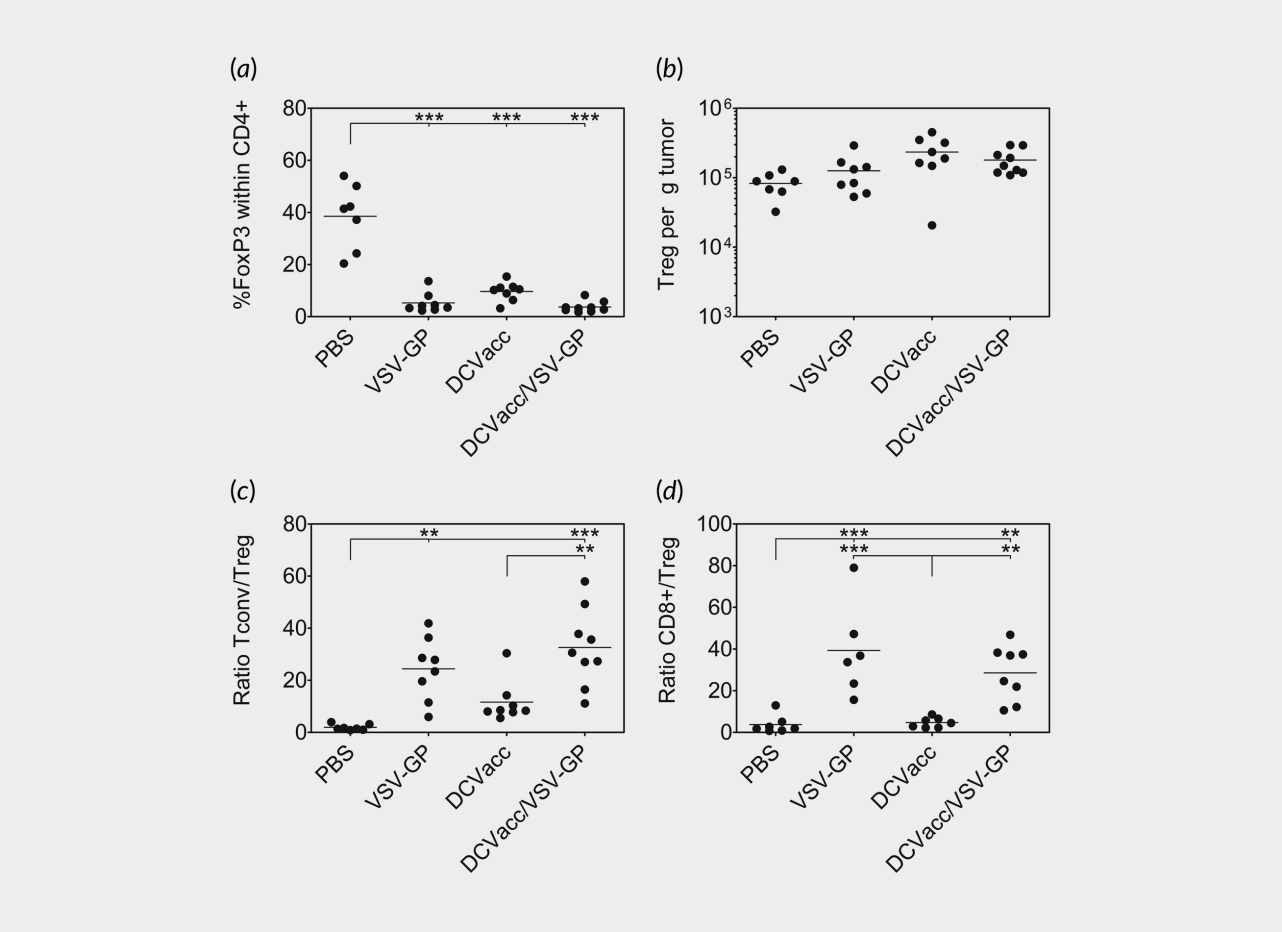
Elevated Tconv/Treg and CD8/Treg ratios in VSV‐GP treated mice. B16‐OVA melanoma in C57BL/6 mice were treated i.t./p.t. on Days 11 and 18 posttransplantation with PBS (control), VSV‐GP (6 × 10^7^ PFU), DCVacc (2 × 10^5^ OVA‐loaded CpG‐matured bmDCs) or DCVacc/VSV‐GP. Seven days after the second treatment cells from B16‐OVA tumors were isolated and CD4/FoxP3 positive cells were analyzed by FACS. Percentage and total cell numbers per g tumor were determined for CD4/FoxP3 double‐positive Tregs. Ratios for FoxP3 negative conventional CD4 T cells and Tregs (Tconv/Treg) as well as for CD8 T cells and Tregs (CD8/Treg) were also calculated. Data represent results from two independent experiments. Data were analyzed by ANOVA followed by Tukey's multiple comparisons test (*p ≤ 0.05,**p ≤ 0.01,***p < 0.001).

Tregs can suppress the antitumoral immune response and it has been recently shown that high CD8 T cell/Treg ratios are associated with improved survival.^25^, ^26^ DCVacc, VSV‐GP and DCVacc/VSV‐GP treatments all resulted in a significant reduction of FoxP3+ regulatory T cells (Treg) within the CD4 T cell compartment compared to the PBS control group (Fig. 5 *a*). Nevertheless, absolute numbers of Tregs did not show any significant differences between the treatment groups (Fig. 5 *b*). However, the FoxP3 negative conventional helper CD4 T cells (Tconv)/Treg ratios within the tumor were significantly elevated upon oncolytic VSV‐GP therapy compared to the PBS control group. This was also the case when DCVacc/VSV‐GP treatment was compared to the PBS or DCVacc groups (Fig. 5 *c*). We also detected a strong increase in CD8 T cell/Treg ratios within the tumor after treatment with either VSV‐GP or DCVacc/VSV‐GP (Fig. 5 *d*). Of note, although NK cells were demonstrated to be important for DC vaccinations, we did not observe any significant changes in total NK cells numbers in the tumor tissue upon any of the treatments (Supporting Information Fig. S6). In addition, NK cell depletion was performed, since NK cells are also known to contribute to the therapeutic efficacy of both DC‐based cancer vaccines and VSV.^27^, ^28^ We depleted either CD8 T cells or NK cells alone or together during DCVacc/VSV‐GP treatments. The CD8 T cells, as well as NK cells, were efficiently depleted via i.p. injection of monoclonal antibodies (Supporting Information Fig. S7). Whereas depletion of NK cells upon DCVacc/VSV‐GP treatment did not significantly influence survival of mice, CD8 T cell depletion significantly reduced treatment efficacy (Supporting Information Fig. S8). Depletion of NK cells together with CD8 T cells did not further influence survival.

Thus, our analysis of DCVacc/VSV‐GP treated tumors revealed an inflammatory response characterized by an infiltration of both CD4 and CD8 T cells resulting in a significant increase in both Tconv/Treg and CD8 T cells/Treg ratios, relative to the DCvacc treated animals. This could be one explanation of why the combination therapy was so much more effective than the DCvacc treatment, despite the similar level of OVA‐specific T cells in both treatment groups.

We also studied lymphocyte infiltration in mice carrying bilateral B16‐OVA tumors. Again, the combination of DCVacc/VSV‐GP, as well as the VSV‐GP and DCVacc single treatments resulted in significantly increased numbers of TILs in the treated tumor, compared to the control group (Supporting Information Fig. S9*A*). Similarly, in contralateral, nontreated tumors, the number of TILs was significantly elevated in all three treatment regimens relative to the PBS treated group (Supporting Information Fig. S9*B*). DCVacc/VSV‐GP treatments resulted in a significant elevation in both Tconv/Treg and CD8 T cell/Treg ratios not only in the treated tumors (Supporting Information Fig. S9*A*) but also in the contralateral nontreated tumors (Supporting Information Fig. S9*B*). These data show that all treatments induced a systemic antitumoral immune response.

### VSV‐GP alone or in combination with DCVacc induces an early inflammatory response in tumor tissue

**Figure 6:**
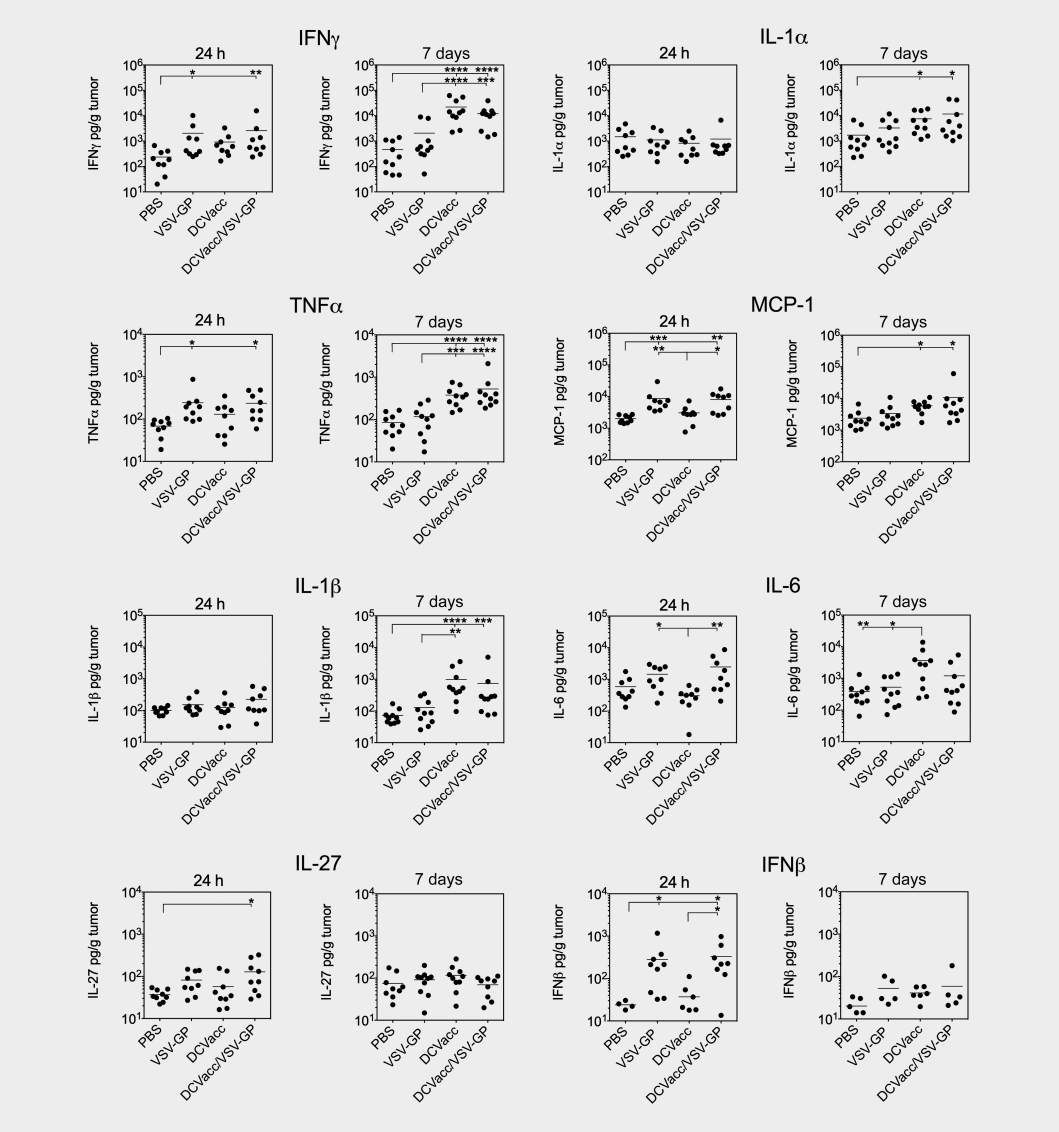
Cytokine profile in tumors after VSV‐GP and DCVacc single and combination treatments. B16‐OVA melanoma in C57BL/6 mice were treated i.t./p.t. on Days 11 posttransplantation with PBS (control), VSV‐GP (6 × 10^7^ PFU), DCVacc (2 × 10^5^ OVA‐loaded CpG‐matured bmDCs) or DCVacc/VSV‐GP. Cytokines were determined in tumor lysates 24 hr and 7 days posttreatments. Data represent results of two independent experiments. Data were analyzed by ANOVA followed by Tukey's multiple comparisons test (*p ≤ 0.05,**p ≤ 0.01,***p < 0.001,****p < 0.0001).

Since cytokines play an important role in the regulation of the tumor microenvironment, we measured the inflammatory cytokines in tumor lysates 24 hr and 7 days after VSV‐GP and DCVacc single and combination therapies using the LEGENDplex™ technology (Fig. 6). We found that VSV‐GP and DCVacc/VSV‐GP induced the release of the cytokines IFNγ, TNFα, MCP‐1 and IL‐6 as early as 24 hr posttreatment. As expected VSV‐GP alone or in combination with DCVacc significantly induced IFNβ production. We also measured the amount of IL‐28 by ELISA, which was also significantly elevated 24 hr after VSV‐GP treatment (Supporting Information Fig. S10). Seven days posttreatment, DCVacc alone or in combination with VSV‐GP significantly elevated the levels of IFNγ, TNFα, IL‐1α, IL‐1β, MCP‐1 and IL‐6.

## Discussion

In previous preclinical studies, we demonstrated that VSV‐GP is a potent and safe OV.^14^, ^15^, ^16^ We here report that the combination of VSV‐GP virotherapy with a DC‐based cancer vaccine significantly improved therapeutic efficacy over the single treatments in the syngeneic transplantable B16‐OVA melanoma model. Improved therapeutic efficacy of the combination therapy was found to be associated with an increased infiltration of activated CD8 T cells into tumor tissue that resulted in a drastic increase in the ratio of activated to regulatory T lymphocytes.

OVs lyse cancer cells whereby cancer tissue is destroyed and cancer antigens are set free in an inflamed environment, which they have the potential to stimulate an anticancer immune response.^17^ Thus, a strong viral oncolysis can *per se* enhance the efficacy of a second cancer treatment. However, as already described for VSV,^21^ VSV‐GP replicated in only a minority of cells in the B16‐OVA tumor and only during the first days. However, albeit the limited direct oncolysis, cytokines like IFNγ, TNFα and IFNβ, induced in the tumor tissue early after VSV‐GP treatment, are known to be able to induce apoptosis and may be involved in the increased caspase‐3 staining seen in the tumor tissue.

In line with our results demonstrating weak OVA‐specific CD8 T cell responses after VSV‐GP treatment, Leveille *et al*. could also not detect a significant tumor‐specific T cell immune response upon VSV‐infection of B16‐OVA melanoma, which was explained by direct infection and destruction of tumor‐associated DCs.^29^ As VSV‐GP infects DCs less efficiently than VSV, VSV‐GP may be more potent in the induction of an anticancer immune response than VSV, although, still not effective enough to induce remission in the majority of tumors.^14^ In contrast to the weak antitumor immune response, a potent antiviral immune response was found, which may have competed with the induction of an antitumor immune response induced by viral oncolysis, thereby limiting the cancer vaccination effect of VSV‐GP.^30^

Nevertheless, in combination with a DC‐vaccine, VSV‐GP was very efficient in controlling tumor growth. DC‐based vaccination has its limitations in cancer therapy due to diminished DC functions and reduced antigen presentation within the immunosuppressive tumor microenvironment.^31^, ^32^ In line with these observations, the DCVacc monotherapy was not very effective in the B16‐OVA melanoma model, despite the potent activation of tumor‐specific CTLs. Secretion of immunosuppressive cytokines such as TGFβ, IL‐6 and IL‐10 by tumor, stroma and inflammatory cells have been shown to stimulate Treg proliferation and reduce the efficacy of cancer vaccine.^33^, ^34^ Accordingly, neither Tconv/Treg nor CD8/Treg ratios in tumor tissues were significantly changed by DCVacc treatment, implying that DCVacc did not overcome the immune evasion of the B16‐OVA tumor.

Previous investigations^17^, ^28^, ^35^, ^36^, ^37^ suggest that OV therapy in cancer tissue can overcome local immune suppression. Thus, in a combination setting, VSV‐GP would provide an inflammatory environment that acts as an adjuvant for the DCVacc‐induced antitumor CTL responses. Indeed, VSV‐GP alone and in combination with DCVacc induced cytokines like IFNγ, TNFα, MCP‐1 and IL‐6 already 24 hr after the treatment, which are expected to support the development of an inflammatory TME. However, VSV‐GP alone only transiently increased cytokines in the TME. In contrast, DCVacc either alone or in combination with VSV‐GP had a delayed effect on cytokines. However, the early and prolonged stimulation of inflammatory cytokines after DCVacc/VSV‐GP treatment did not translate into an increase in the number of OVA‐specific CD8 T cells relative to DCVacc single treatment.

Since an increased OVA‐specific CD8 T cell response in the combination group did not explain the therapeutic efficacy of the DCVacc/VSV‐GP treatment, we focused on the number and composition of leukocytes found in tumors, which has been shown to correlate with tumor prognosis.^25^, ^26^, ^38^, ^39^, ^40^, ^41^ Indeed, TILs, such as CD4 and highly activated CD8 T cells, were increased upon DCVacc/VSV‐GP treatment and were mainly directed against VSV‐GP. In addition, we analyzed Treg levels in the tumor as the effector CD8/regulatory T cell ratios are known to be a potent prognostic factor for the efficacy of immunotherapies in melanoma.^25^, ^26^, ^42^, ^43^, ^44^ Notably, the DCVacc/VSV‐GP treatment did not influence the numbers of Tregs in the tumor, but the VSV‐GP related lymphocyte infiltration, mainly with activated virus‐specific CD8 T cells, drastically increased the effector T cell/Treg ratios. Interestingly, in the tumor tissue, neither the total number of NK cells nor of myeloid‐derived suppressor cells (MDSC; data not shown) were altered upon DCVacc/VSV‐GP treatment relative to DCVacc single treatment. Remarkably, pronounced infiltration of lymphocytes was also observed in contralateral (nontreated) tumors in both monotherapies and the combinational treatment. Furthermore, the DCVacc/VSV‐GP combination led to significantly elevated Tconv/Treg and CD8/Treg ratios also in contralateral tumors, demonstrating that the local combination therapy generated systemic immune responses. Thus, local DCVacc/VSV‐GP treatment could be effective in metastatic disease in the clinic.

Recent studies showed that the efficacy of oncolytic virotherapy as well as of DC‐based vaccination depends on the induction of specific effector T cell subpopulations as well as the activation of NK cells.^27^, ^36^ Particularly IL‐28 dependent NK cell functions have been demonstrated to be important for efficacy of VSV in B16‐OVA model.^28^ Interestingly, DCVacc/VSV‐GP did not result in an increased IL‐28 production in the tumor tissue. In our study, the improved therapeutic effect of the DCVacc/VSV‐GP combination therapy was dependent mainly on CD8 T cells and not NK cells. DC‐based vaccination alone has been shown to depend on NK cell activation to provide cytokines such as IFNγ that induce a Th1/CTL response.^27^ In the combination therapy, however, local infection of tumor tissue by VSV‐GP induced type‐I IFN, and thereby a Th1 switch.^45^ This occurred even in the absence of a DC/NK cell cross‐talk, which could be the reason that NK cells are not essential for the therapeutic efficacy of the combination treatment.

B16‐OVA melanoma represents a well‐established model to test novel concepts in cancer immunotherapies. Although DCVacc and VSV‐GP therapies acted synergistically in B16‐OVA melanoma, other tumor models like the transformed lung epithelia TC‐1, the colon adenocarcinoma MC38, the lung carcinoma LLC, and the melanoma B16 in B6 mice might be tested. Beside model antigens like the HPV E6/E7 in TC‐1 and the β‐galactosidase in CT26, additional models would also allow us to test therapeutic efficacy of DCVacc/VSV‐GP for “real” tumor antigens like the gp100/Trp2 antigens in B16, the survivin in LLC or the Adpgk1 in MC38. Furthermore, these tumor models differ in their susceptibility for VSV‐GP, which due to differences in the oncolytic activity of VSV‐GP might also influence therapeutic outcome of VSV‐GP and DCVacc/VSV‐GP treatments. As our results suggest that VSV‐GP improves DCVacc by modulating the TME, immunological “hot” and “cold” tumor models might also differ in their response to both VSV‐GP single and DCVacc/VSV‐GP combination treatments. Moreover, different treatment schedules and the addition of other cancer drugs could be tested in these models, as the immunotherapies are evolving to become combination therapies. In the clinical setting, immune checkpoint inhibitors (ICI) will most likely be applied together with a potential combination of OVs and cancer vaccines as described here.

Taken together, the oncolytic and immune‐stimulatory effects of VSV‐GP synergize with the DCVacc for the efficient induction of a tumor‐specific T cell response. DC‐based vaccinations already proved to be safe and well tolerated in patients^46^ and VSV‐GP has been shown to be a safe and efficient oncolytic agent in a number of animal models.^14^, ^15^, ^47^ Thus, the data of this study could provide a basis for the translation of the DCVacc/VSV‐GP combination therapy into clinical testing.

## Supporting information


**Appendix S1**: Supporting information

## References

[1] Chen DS , Mellman I . Elements of cancer immunity and the cancer‐immune set point. Nature 2017;541:321–30. 28102259 10.1038/nature21349

[2] Palucka K , Banchereau J . Dendritic‐cell‐based therapeutic cancer vaccines. Immunity 2013;39:38–48. 23890062 10.1016/j.immuni.2013.07.004 PMC3788678

[3] Gross S , Erdmann M , Haendle I , et al. Twelve‐year survival and immune correlates in dendritic cell‐vaccinated melanoma patients. JCI Insight 2017;2:e91438. 10.1172/jci.insight.91438 PMC5396520 28422751

[4] Garg AD , Coulie PG , Van den Eynde BJ , et al. Integrating next‐generation dendritic cell vaccines into the current cancer immunotherapy landscape. Trends Immunol 2017;38:577–93. 28610825 10.1016/j.it.2017.05.006

[5] Morse MA , Coleman RE , Akabani G , et al. Migration of human dendritic cells after injection in patients with metastatic malignancies. Cancer Res 1999;59:56–8. 9892184

[6] Quillien V , Moisan A , Carsin A , et al. Biodistribution of radiolabelled human dendritic cells injected by various routes. Eur J Nucl Med Mol Imaging 2005;32:731–41. 15924229 10.1007/s00259-005-1825-9

[7] Steinman RM , Nussenzweig MC . Avoiding horror autotoxicus: the importance of dendritic cells in peripheral T cell tolerance. Proc Natl Acad Sci USA 2002;99:351–8. 11773639 10.1073/pnas.231606698 PMC117564

[8] Buschow SI , Ramazzotti M , Reinieren‐Beeren IMJ , et al. Survival of metastatic melanoma patients after dendritic cell vaccination correlates with expression of leukocyte phosphatidylethanolamine‐binding protein 1/Raf kinase inhibitory protein. Oncotarget 2017;8:67439–56. 28978044 10.18632/oncotarget.18698 PMC5620184

[9] Ellebaek E , Engell‐Noerregaard L , Iversen TZ , et al. Metastatic melanoma patients treated with dendritic cell vaccination, Interleukin‐2 and metronomic cyclophosphamide: results from a phase II trial. Cancer Immunol Immunother 2012;61:1791–804. 22426890 10.1007/s00262-012-1242-4 PMC11029126

[10] Romani N , Thurnher M , Idoyaga J , et al. Targeting of antigens to skin dendritic cells: possibilities to enhance vaccine efficacy. Immunol Cell Biol 2010;88:424–30. 20368713 10.1038/icb.2010.39 PMC2907485

[11] Schuler‐Thurner B , Schultz ES , Berger TG , et al. Rapid induction of tumor‐specific type 1 T helper cells in metastatic melanoma patients by vaccination with mature, cryopreserved, peptide‐loaded monocyte‐derived dendritic cells. J Exp Med 2002;195:1279–88. 12021308 10.1084/jem.20012100 PMC2193752

[12] Tripp CH , Ebner S , Ratzinger G , et al. Conditioning of the injection site with CpG enhances the migration of adoptively transferred dendritic cells and endogenous CD8+ T‐cell responses. J Immunother 2010;33:115–25. 20145551 10.1097/CJI.0b013e3181b8ef5f

[13] Motz GT , Coukos G . Deciphering and reversing tumor immune suppression. Immunity 2013;39:61–73. 23890064 10.1016/j.immuni.2013.07.005 PMC3782392

[14] Muik A , Kneiske I , Werbizki M , et al. Pseudotyping vesicular stomatitis virus with lymphocytic choriomeningitis virus glycoproteins enhances infectivity for glioma cells and minimizes neurotropism. J Virol 2011;85:5679–84. 21450833 10.1128/JVI.02511-10 PMC3094995

[15] Muik A , Stubbert LJ , Jahedi RZ , et al. Re‐engineering vesicular stomatitis virus to abrogate neurotoxicity, circumvent humoral immunity, and enhance oncolytic potency. Cancer Res 2014;74:3567–78. 24812275 10.1158/0008-5472.CAN-13-3306

[16] Tober R , Banki Z , Egerer L , et al. VSV‐GP: a potent viral vaccine vector that boosts the immune response upon repeated applications. J Virol 2014;88:4897–907. 24554655 10.1128/JVI.03276-13 PMC3993835

[17] Guo ZS , Liu Z , Bartlett DL . Oncolytic immunotherapy: dying the right way is a key to eliciting potent antitumor immunity. Front Oncol 2014;4:74. 24782985 10.3389/fonc.2014.00074 PMC3989763

[18] Prestwich RJ , Errington F , Ilett EJ , et al. Tumor infection by oncolytic reovirus primes adaptive antitumor immunity. Clin Cancer Res 2008;14:7358–66. 19010851 10.1158/1078-0432.CCR-08-0831 PMC2701231

[19] Sobol PT , Boudreau JE , Stephenson K , et al. Adaptive antiviral immunity is a determinant of the therapeutic success of oncolytic virotherapy. Mol Ther 2011;19:335–44. 21119618 10.1038/mt.2010.264 PMC3034857

[20] Melzer MK , Lopez‐Martinez A , Altomonte J . Oncolytic vesicular stomatitis virus as a Viro‐immunotherapy: defeating cancer with a "hammer" and "anvil". Biomedicine 2017;5:8. 10.3390/biomedicines5010008 PMC5423493 28536351

[21] Galivo F , Diaz RM , Wongthida P , et al. Single‐cycle viral gene expression, rather than progressive replication and oncolysis, is required for VSV therapy of B16 melanoma. Gene Ther 2010;17:158–70. 20016540 10.1038/gt.2009.161 PMC3934361

[22] Brown DM , Fisher TL , Wei C , et al. Tumours can act as adjuvants for humoral immunity. Immunology 2001;102:486–97. 11328383 10.1046/j.1365-2567.2001.01213.x PMC1783199

[23] Lutz MB , Kukutsch N , Ogilvie AL , et al. An advanced culture method for generating large quantities of highly pure dendritic cells from mouse bone marrow. J Immunol Methods 1999;223:77–92. 10037236 10.1016/s0022-1759(98)00204-x

[24] Galon J , Costes A , Sanchez‐Cabo F , et al. Type, density, and location of immune cells within human colorectal tumors predict clinical outcome. Science 2006;313:1960–4. 17008531 10.1126/science.1129139

[25] Gooden MJ , de Bock GH , Leffers N , et al. The prognostic influence of tumour‐infiltrating lymphocytes in cancer: a systematic review with meta‐analysis. Br J Cancer 2011;105:93–103. 21629244 10.1038/bjc.2011.189 PMC3137407

[26] Sato E , Olson SH , Ahn J , et al. Intraepithelial CD8+ tumor‐infiltrating lymphocytes and a high CD8+/regulatory T cell ratio are associated with favorable prognosis in ovarian cancer. Proc Natl Acad Sci USA 2005;102:18538–43. 16344461 10.1073/pnas.0509182102 PMC1311741

[27] Bouwer AL , Saunderson SC , Caldwell FJ , et al. NK cells are required for dendritic cell‐based immunotherapy at the time of tumor challenge. J Immunol 2014;192:2514–21. 24477907 10.4049/jimmunol.1202797

[28] Wongthida P , Diaz RM , Galivo F , et al. Type III IFN interleukin‐28 mediates the antitumor efficacy of oncolytic virus VSV in immune‐competent mouse models of cancer. Cancer Res 2010;70:4539–49. 20484025 10.1158/0008-5472.CAN-09-4658 PMC3896099

[29] Leveille S , Goulet ML , Lichty BD , et al. Vesicular stomatitis virus oncolytic treatment interferes with tumor‐associated dendritic cell functions and abrogates tumor antigen presentation. J Virol 2011;85:12160–9. 21917977 10.1128/JVI.05703-11 PMC3209377

[30] Galivo F , Diaz RM , Thanarajasingam U , et al. Interference of CD40L‐mediated tumor immunotherapy by oncolytic vesicular stomatitis virus. Hum Gene Ther 2010;21:439–50. 19922169 10.1089/hum.2009.143 PMC2865217

[31] Dhodapkar MV , Krasovsky J , Steinman RM , et al. Mature dendritic cells boost functionally superior CD8(+) T‐cell in humans without foreign helper epitopes. J Clin Invest 2000;105:R9–R14. 10727452 10.1172/JCI9051 PMC377466

[32] Dhodapkar MV , Steinman RM , Sapp M , et al. Rapid generation of broad T‐cell immunity in humans after a single injection of mature dendritic cells. J Clin Invest 1999;104:173–80. 10411546 10.1172/JCI6909 PMC408478

[33] Maldonado RA , von Andrian UH . How tolerogenic dendritic cells induce regulatory T cells. Adv Immunol 2010;108:111–65. 21056730 10.1016/B978-0-12-380995-7.00004-5 PMC3050492

[34] Zou W . Immunosuppressive networks in the tumour environment and their therapeutic relevance. Nat Rev Cancer 2005;5:263–74. 15776005 10.1038/nrc1586

[35] Altomonte J , Wu L , Chen L , et al. Exponential enhancement of oncolytic vesicular stomatitis virus potency by vector‐mediated suppression of inflammatory responses in vivo. Mol Ther 2008;16:146–53. 18071337 10.1038/sj.mt.6300343 PMC2930752

[36] Diaz RM , Galivo F , Kottke T , et al. Oncolytic immunovirotherapy for melanoma using vesicular stomatitis virus. Cancer Res 2007;67:2840–8. 17363607 10.1158/0008-5472.CAN-06-3974

[37] Wu L , Huang TG , Meseck M , et al. rVSV(M Delta 51)‐M3 is an effective and safe oncolytic virus for cancer therapy. Hum Gene Ther 2008;19:635–47. 18533893 10.1089/hum.2007.163 PMC2775926

[38] Ladanyi A , Somlai B , Gilde K , et al. T‐cell activation marker expression on tumor‐infiltrating lymphocytes as prognostic factor in cutaneous malignant melanoma. Clin Cancer Res 2004;10:521–30. 14760073 10.1158/1078-0432.ccr-1161-03

[39] Galon J , Pages F , Marincola FM , et al. Cancer classification using the immunoscore: a worldwide task force. J Transl Med 2012;10:205. 23034130 10.1186/1479-5876-10-205 PMC3554496

[40] Leffers N , Gooden MJ , de Jong RA , et al. Prognostic significance of tumor‐infiltrating T‐lymphocytes in primary and metastatic lesions of advanced stage ovarian cancer. Cancer Immunol Immunother 2009;58:449–59. 18791714 10.1007/s00262-008-0583-5 PMC11030692

[41] Zhang L , Conejo‐Garcia JR , Katsaros D , et al. Intratumoral T cells, recurrence, and survival in epithelial ovarian cancer. N Engl J Med 2003;348:203–13. 12529460 10.1056/NEJMoa020177

[42] Curran MA , Montalvo W , Yagita H , et al. PD‐1 and CTLA‐4 combination blockade expands infiltrating T cells and reduces regulatory T and myeloid cells within B16 melanoma tumors. Proc Natl Acad Sci USA 2010;107:4275–80. 20160101 10.1073/pnas.0915174107 PMC2840093

[43] Ladoire S , Senovilla L , Enot D , et al. Biomarkers of immunogenic stress in metastases from melanoma patients: correlations with the immune infiltrate. Oncoimmunology 2016;5:e1160193. 27471635 10.1080/2162402X.2016.1160193 PMC4938307

[44] Quezada SA , Peggs KS , Curran MA , et al. CTLA4 blockade and GM‐CSF combination immunotherapy alters the intratumor balance of effector and regulatory T cells. J Clin Invest 2006;116:1935–45. 16778987 10.1172/JCI27745 PMC1479425

[45] Fernandez M , Porosnicu M , Markovic D , et al. Genetically engineered vesicular stomatitis virus in gene therapy: application for treatment of malignant disease. J Virol 2002;76:895–904. 11752178 10.1128/JVI.76.2.895-904.2002 PMC136833

[46] Anguille S , Smits EL , Lion E , et al. Clinical use of dendritic cells for cancer therapy. Lancet Oncol 2014;15:e257–67. 24872109 10.1016/S1470-2045(13)70585-0

[47] Dold C , Rodriguez Urbiola C , Wollmann G , et al. Application of interferon modulators to overcome partial resistance of human ovarian cancers to VSV‐GP oncolytic viral therapy. Mol Ther Oncolyt 2016;3:16021. 10.1038/mto.2016.21 PMC5040171 27738655

