# Supplementary material for: Oncolytic virotherapy enhances the efficacy of a cancer vaccine by modulating the tumor microenvironment
Source: Int J Cancer. 2019 Apr 29;145(7):1958–69. doi: 10.1002/ijc.32325 (PMC6767478; doi:10.1002/ijc.32325)
Supplement: Supplementary file 1 — Appendix S1: Supporting information [file IJC-145-1958-s001.doc]

**Supplementary data**

**Supplementary Material and Methods**

**Immunohistological analysis.** Mice were sacrificed either 1 day or 6 days post treatment and tumors were fixed with 4% PFA (Carl Roth, Germany) overnight at 4°C. Tumors were cut in half and equilibrated with 30% sucrose (Merck, Germany) overnight at 4°C. The tumor tissues were embedded into Tissue-Tec O.C.T. compound (Hartenstein, Germany) for cryo-sectioning. The tissues were cut into 10 µm thick slices using a cryostat and slices were thaw-mounted onto polylysine-coated slides and stained for the apoptotic marker active caspase 3 (aCas3), polyclonal rabbit anti-mouse aCas3 (R&D systems, USA) followed by secondary antibody staining with Alexa594-labeled anti-rabbit IgG (ThermoFisher, USA) and nuclear counterstaining with DAPI (Sigma-Aldrich, USA). Immunofluorescent stainings and the VSV-GP distribution (GFP) of the harvested tumor tissues were analyzed using immunofluorescent microscopy.

**Table S1: List of antibodies used for flow cytometric analysis**

| **Molecules** | **Clone** | **Company** |
| --- | --- | --- |
| CD3e-PE | 145-2C11 | BD Bioscience, USA |
| CD3e-PE-Cy5 | 145-2C11 | BD Bioscience, USA |
| CD3e-APC | 145-2C11 | BD Bioscience, USA |
| CD4-FITC | RM4-5 | eBioscience, USA |
| CD4-APC-H7 | GK 1.5 | BD Bioscience, USA |
| CD8a-PE-Cy5 | 53-6.7 | BD Bioscience, USA |
| CD8a-APC-H7 | 53-6.7 | BD Bioscience, USA |
| CD25-APC | PC61.5 | eBioscience, USA |
| CD43-FITC | 1B11 | Biolegend, USA |
| CD45.2-APC | 104 | BD Bioscience, USA |
| CD45-PE-Cy5 | 30-F11 | BD Bioscience, USA |
| FOXP3-PE | FJK-16s | eBioscience, USA |
| IFNγ-FITC | XMG1.2 | BD Bioscience, USA |
| NK1.1-APC-Cy7 | PK136 | BD Bioscience, USA |
| iTAg tetramer/APC-H-2-Kb OVA SIINFEKL |  | MBL, USA |

**
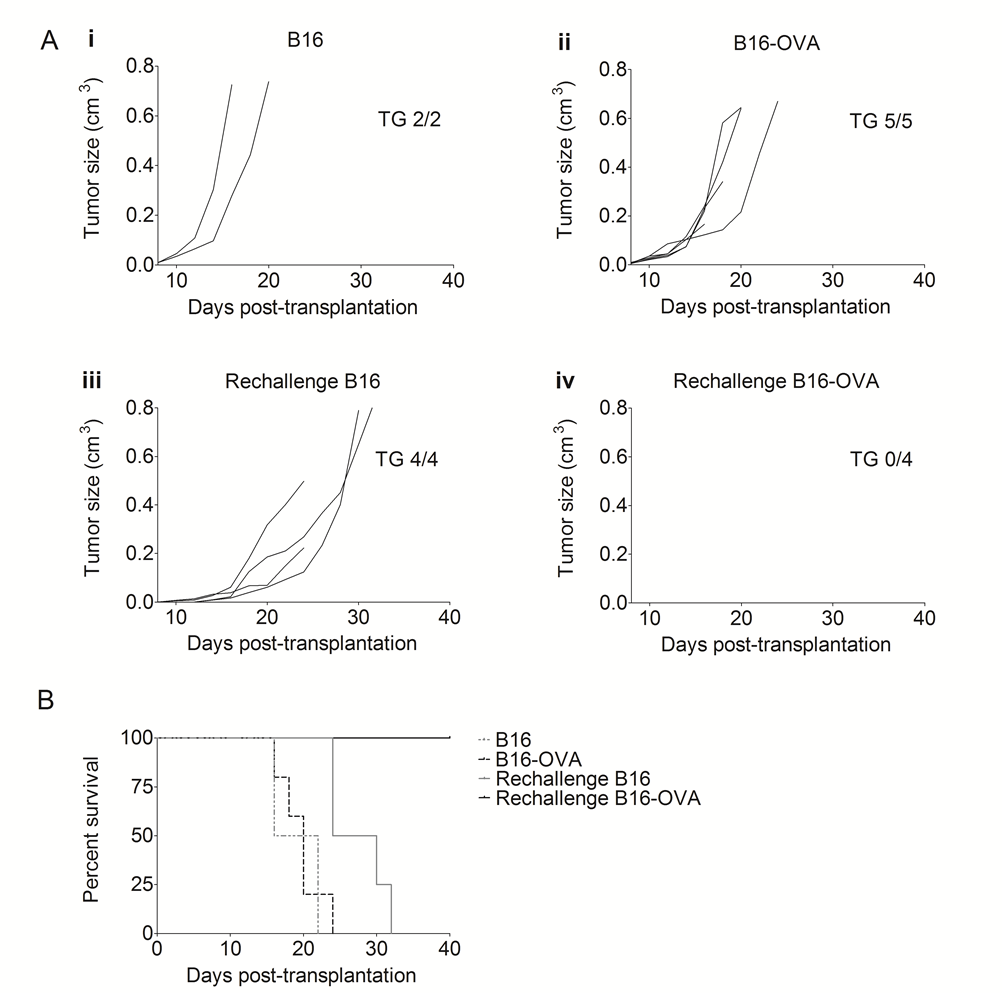
**

**Suppl. Figure 1.** **Re-challenge of cured DCVacc/VSV-GP treated C57BL/6 mice with B16 or B16-OVA.** Control mice were implanted s.c. either with B16 (i) or B16-OVA (ii) cells. Animals showing complete tumor remission after DCVacc/VSV-GP treatments were re-challenged with B16 (iii) or B16-OVA (iv) cells on day 90. (**A**) Tumor growth and (**B**) overall survival were followed up to 40 days post transplantation. Data were analyzed by Mantel-Cox test. B16 vs Rechallenge B16 = p ≤ 0.05; B16 vs Rechallenge B16-OVA = p ≤ 0.05; B16-OVA vs Rechallenge B16 = p ≤ 0.05; B16-OVA vs Rechallenge B16-OVA = p ≤ 0.01 and Rechallenge B16 vs Rechallenge B16-OVA = p ≤ 0.01. Numbers of mice per group and mice with tumor growth (TG) are indicated in the graphs.

**
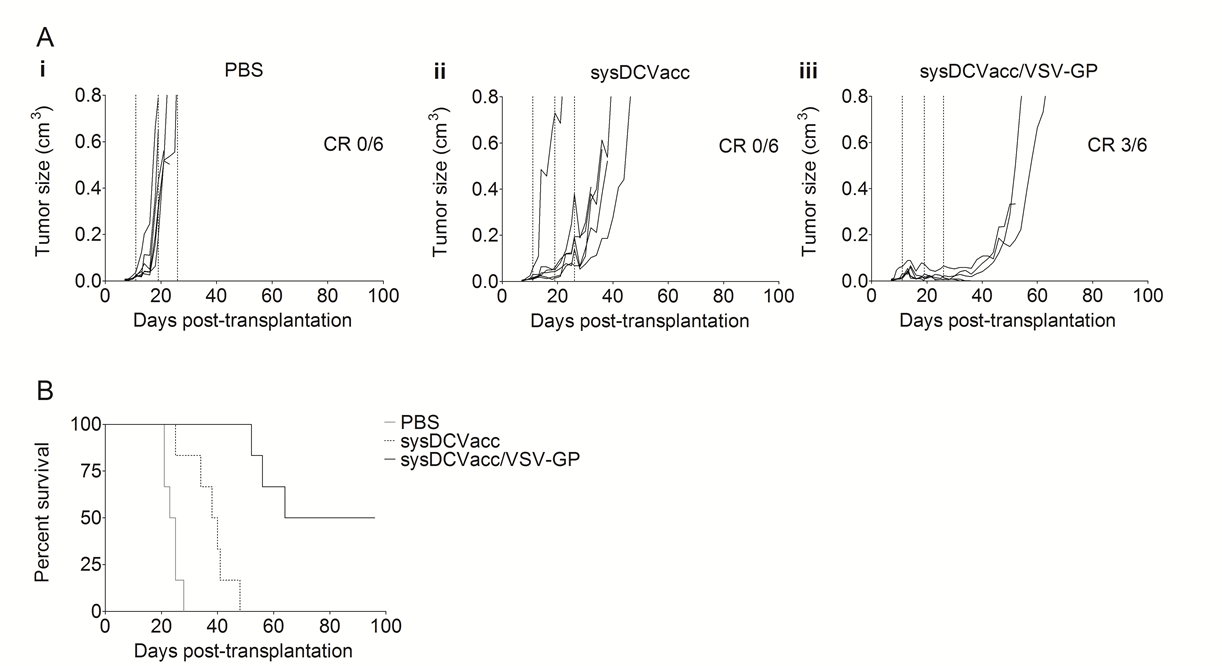
**

**Suppl. Figure 2.** **Systemic DCVacc/VSV-GP combination therapy showed significant survival benefit compared to single systemic DCVacc treatment.** (**A**) Tumor growth curves of B16-OVA melanoma in C57BL/6 mice. DCVacc was applied systemically either alone by administering 2×10^5^ OVA-loaded CpG-matured bmDCs i.v. (sysDCVacc) or i.v. application of sysDCVacc was combined with 6×10^7^ PFU VSV-GP injected i.t./p.t. (sysDCVacc/VSV-GP) on days 11, 18 and 25 post transplantation. PBS injected i.t./p.t. was used as control. (**B**) Overall survival. Data were analyzed by Mantel-Cox test. PBS vs sysDCVacc = p ≤ 0.01, PBS vs sysDCVacc/VSV-GP = p<0.001 and p<0.001 vs sysDCVacc/VSV-GP = p<0.001. Results from one experiment with n=6 mice per group are represented. Mice with a complete response (CR) to the treatment are indicated.

**
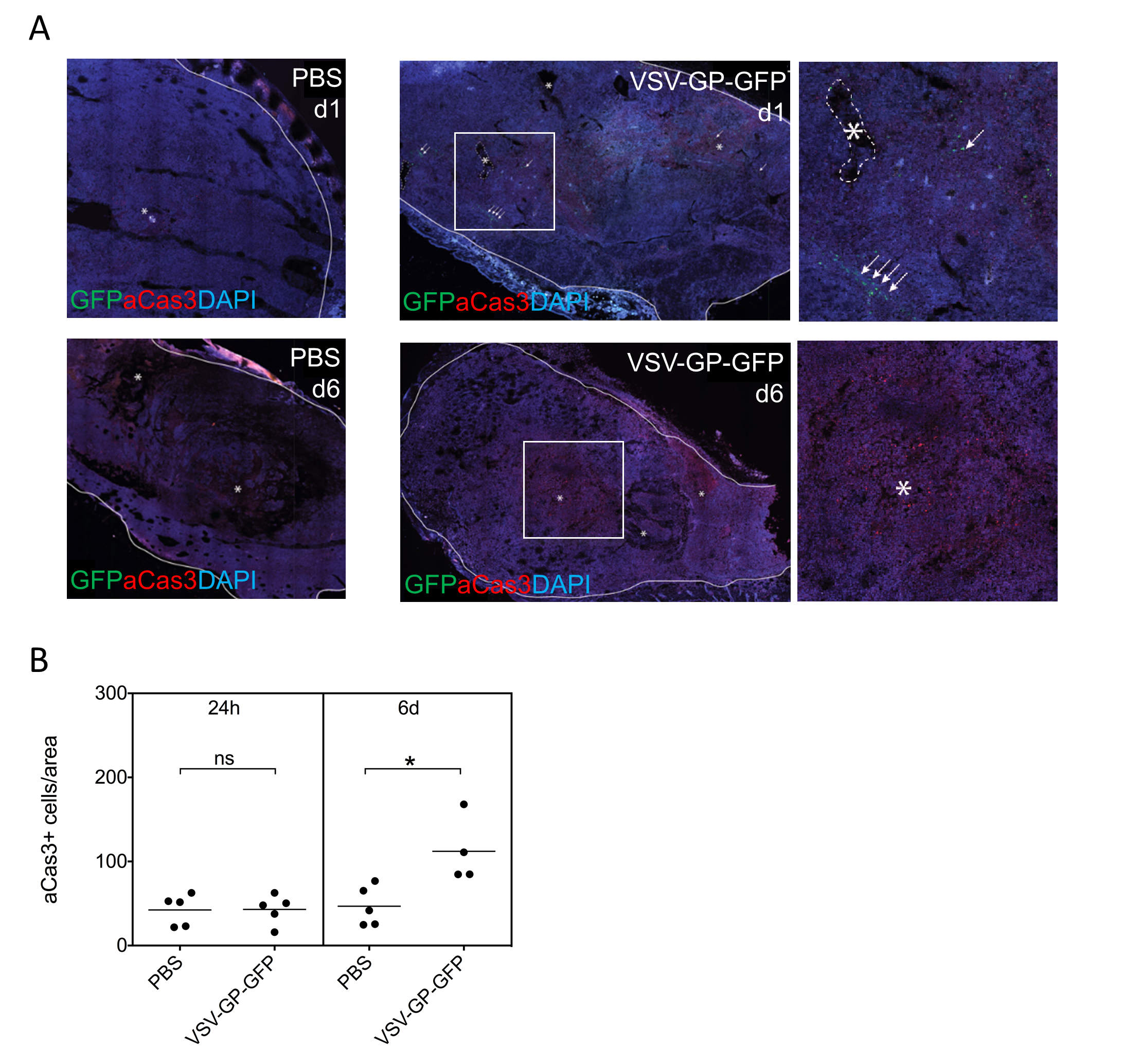
**

**Suppl. Figure 3.** **Transient** **VSV-GP-GFP replication in the B16-OVA tumors.** B16-OVA melanoma were treated i.t. at a tumor size of 0.05-0.15 cm^3^ with PBS (control) or VSV-GP-GFP (5×10^8^ TCID_50_). Tumors were dissected at day 1 (d1) and 6 days (d6) post treatment. **(A)** Immunofluorescent stainings using an anti-caspase3 (aCas3, red) antibody were conducted. DAPI (blue) was used for nuclear counterstain. Dashed lines outline tumor lobules. At day 1, GFP positive cells were detected (white arrows). 6 days after virus application, hardly any GFP positive cells and large areas of aCas3 were detected. Control tumors showed smaller clusters of aCas3 positive cells in close proximity to necrotic areas. Representative images from n=5 mice per group and time point are shown. **(B)** The aCas3+ve cells were counted within four independent areas of each specimen (n=5 tumors/group) for quantification purposes. Data points represent average of aCas3+ve cells per sample and Student´s t-test was performed. Note that number of aCas3+ve cells at d1 is not significantly changed, whereas VSV-GP-treated melanomas showed more aCas3+ve cells compared to controls 6 days following treatment.

**
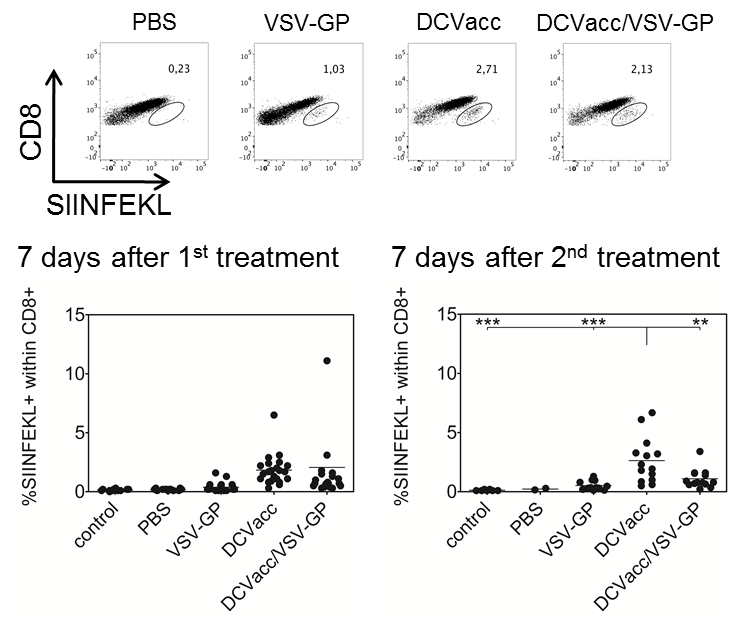
**

**Suppl. Figure 4. Both DCVacc and DCVacc/VSV-GP treatments induced SIINFEKL-specific CD8+ T cell responses in the blood.** B16-OVA melanoma in C57BL/6 mice were treated i.t./p.t. on days 11 and 18 post transplantation with PBS (control), VSV-GP (6×10^7^ PFU), DCVacc (2×10^5^ OVA-loaded CpG-matured bmDCs) or DCVacc/VSV-GP. 7 days after the first and second treatments OVA-specific CD8+ T cell responses were analyzed from the blood using specific tetramers by FACS. FACS dot plots depicting CD8 positive (y-axis) and tetramer positive (x-axis) cells show representative data from the different treatment groups. Data represent cumulative results of 4 independent experiments. Data were analyzed by ANOVA followed by Tukey‘s multiple comparisons test (p≤ 0.01 (**), p<0.001 (***)).

**
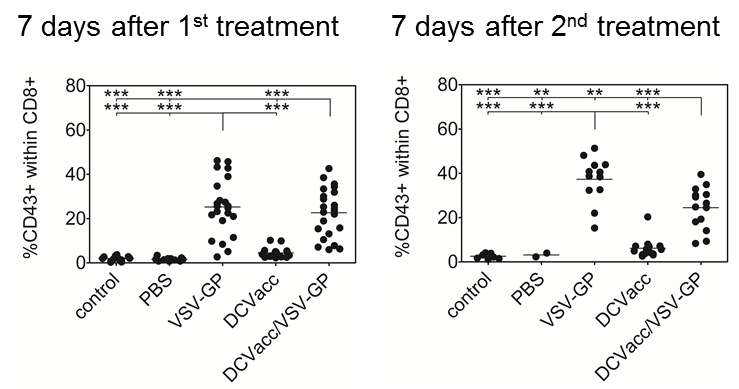
**

**Suppl. Figure 5. Strong activation of CD8 T cells in mice treated with VSV-GP.** B16-OVA melanoma in C57BL/6 mice were treated i.t./p.t. on days 11 and 18 post transplantation with PBS (control), VSV-GP (6×10^7^ PFU), DCVacc (2×10^5^ OVA-loaded CpG-matured bmDCs) or DCVacc/VSV-GP. 7 days after the first and the second treatment, activation of CD8+ T cells was measured by the expression of CD43 from the blood by FACS. Data represent results derived from 4 independent experiments. Data were analyzed by ANOVA followed by Tukey‘s multiple comparisons test (p ≤ 0.01 (**), p<0.0001 (****)).

**
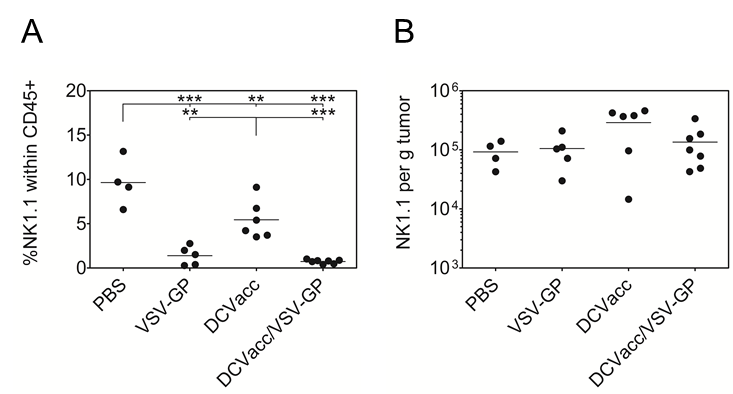
**

**Suppl. Figure 6. NK cells in the tumor tissue.** B16-OVA melanoma in C57BL/6 mice were treated i.t./p.t. at day 11 and 18 post transplantation with PBS (control), VSV-GP (6×10^7^ PFU), DCVacc (2×10^5^ OVA-loaded CpG-matured bmDCs) or DCVacc/VSV-GP. 7 days after the second treatment tumor cells were isolated and TILs were analyzed by FACS. Frequency of NK cells within CD45+ population (**A**) and total cell numbers of CD3+NK1.1+ NK cells per g tumor (**B**) and are shown. Data represent results from 2 independent experiments. Data were analyzed by ANOVA followed by Tukey‘s multiple comparisons test (p ≤ 0.01 (**), p<0.001 (***), p<0.0001 (****)).


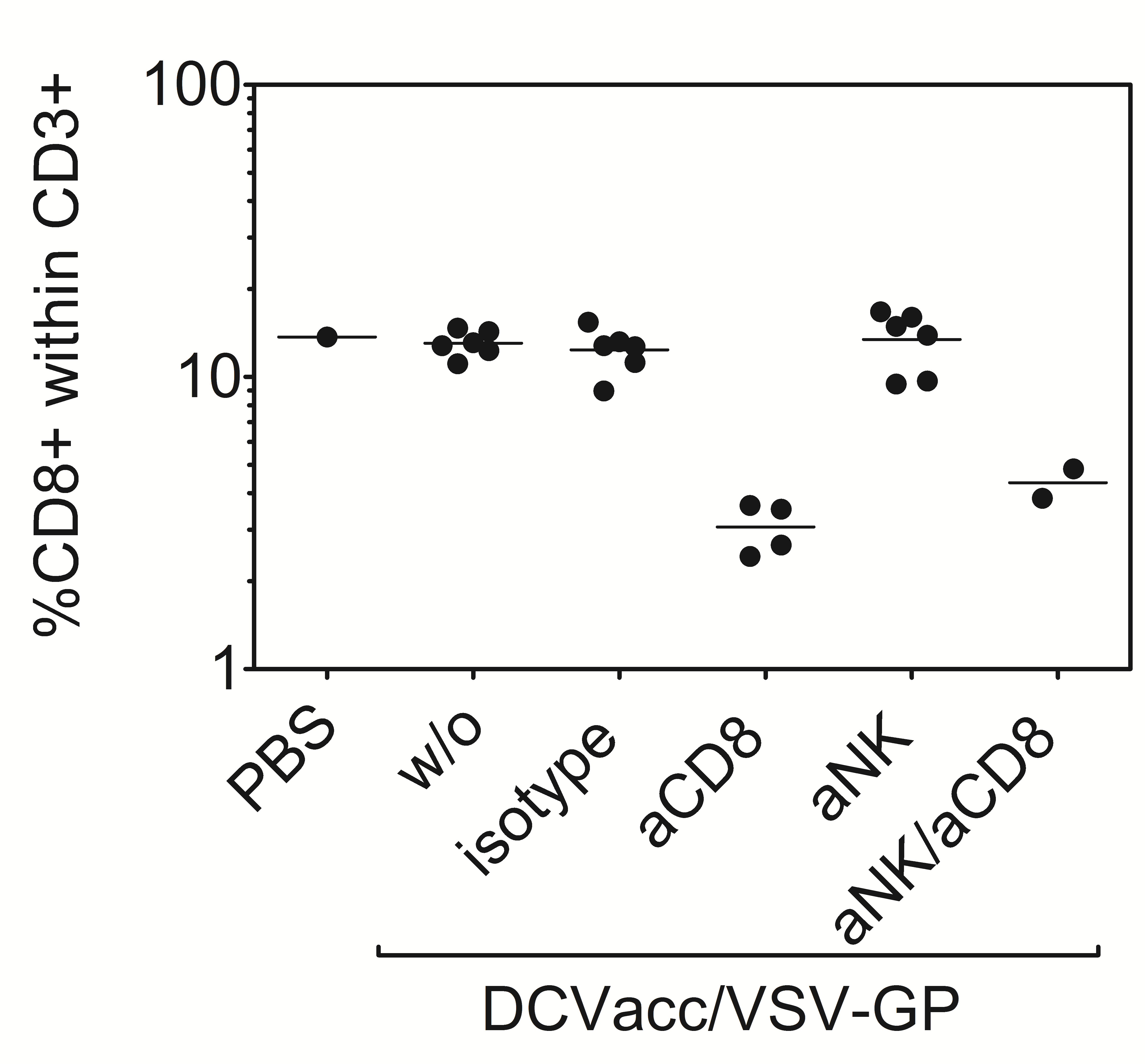

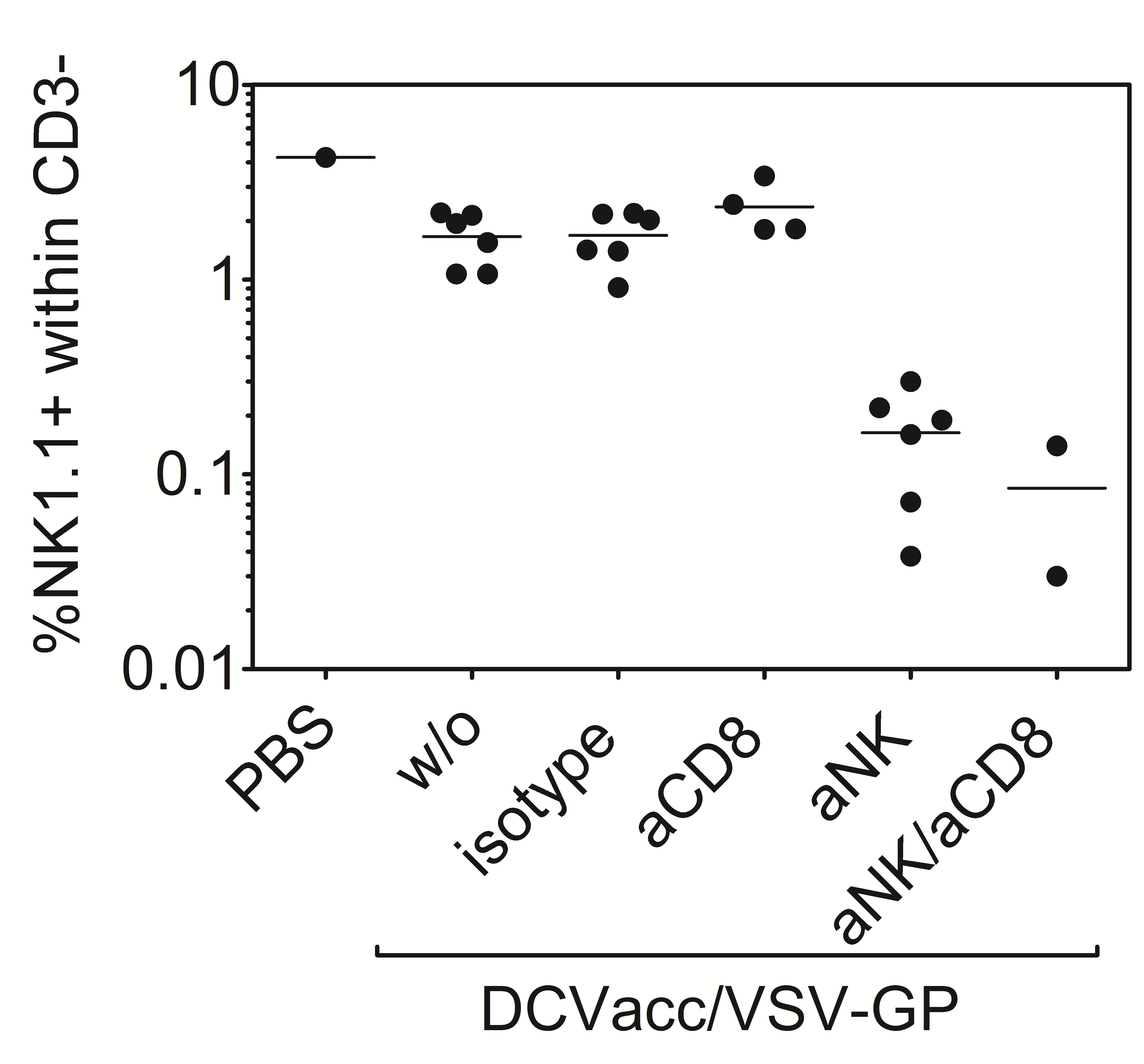

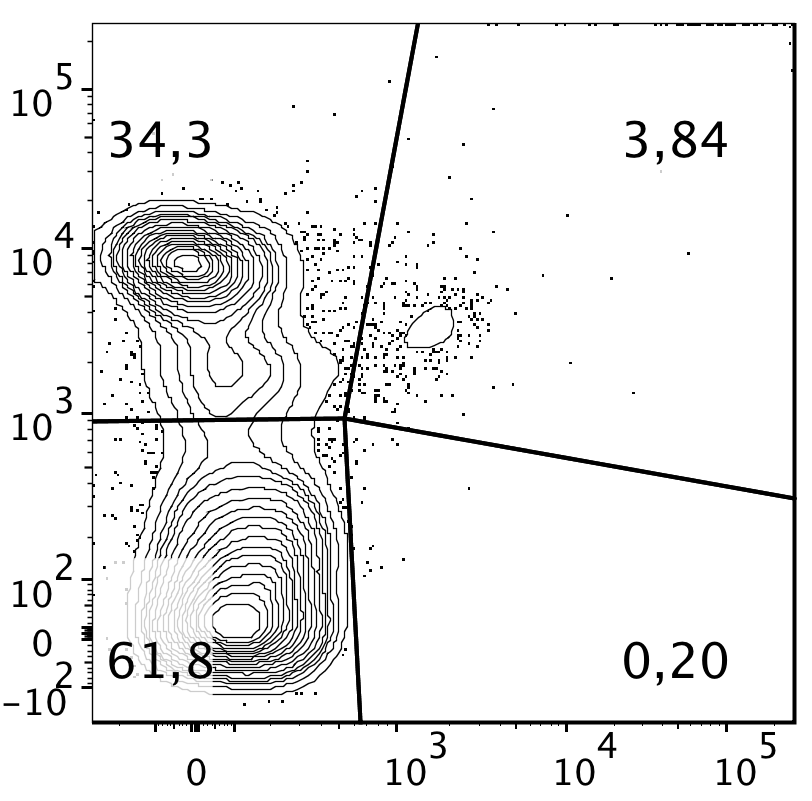

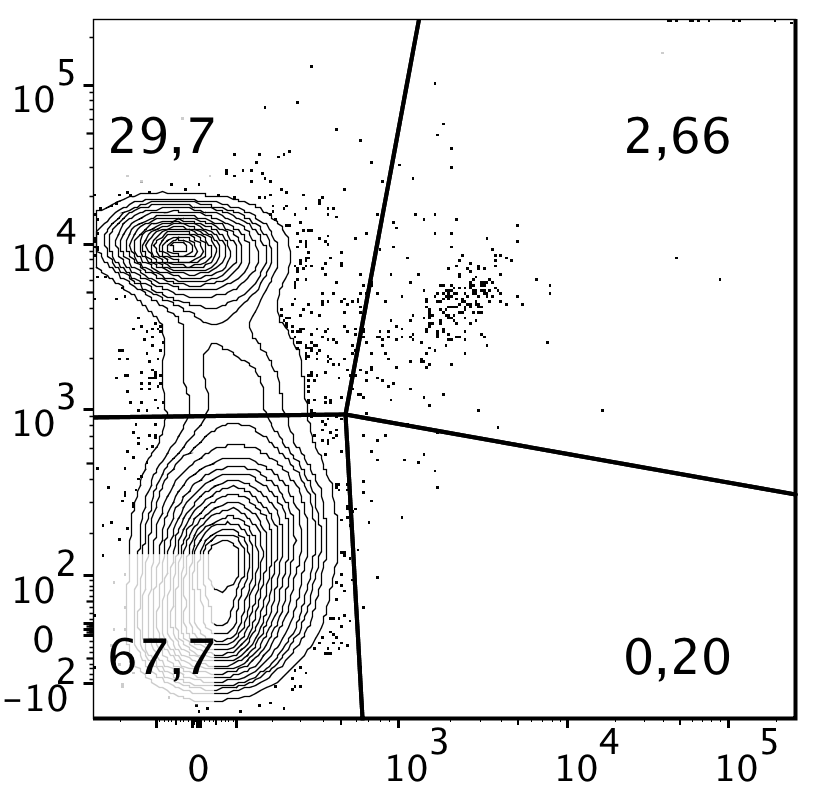

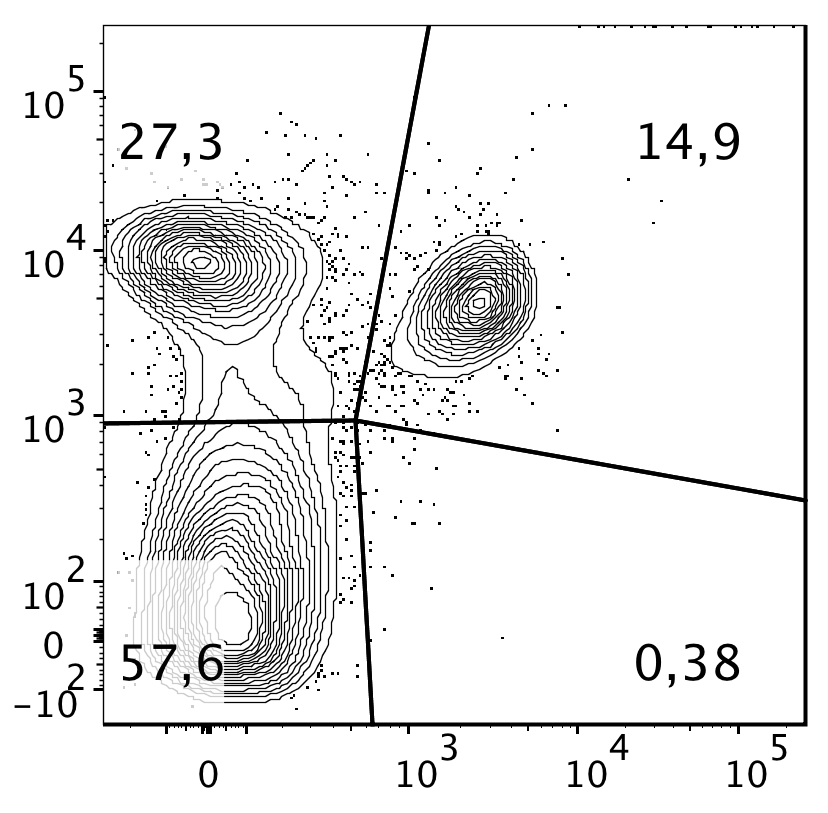

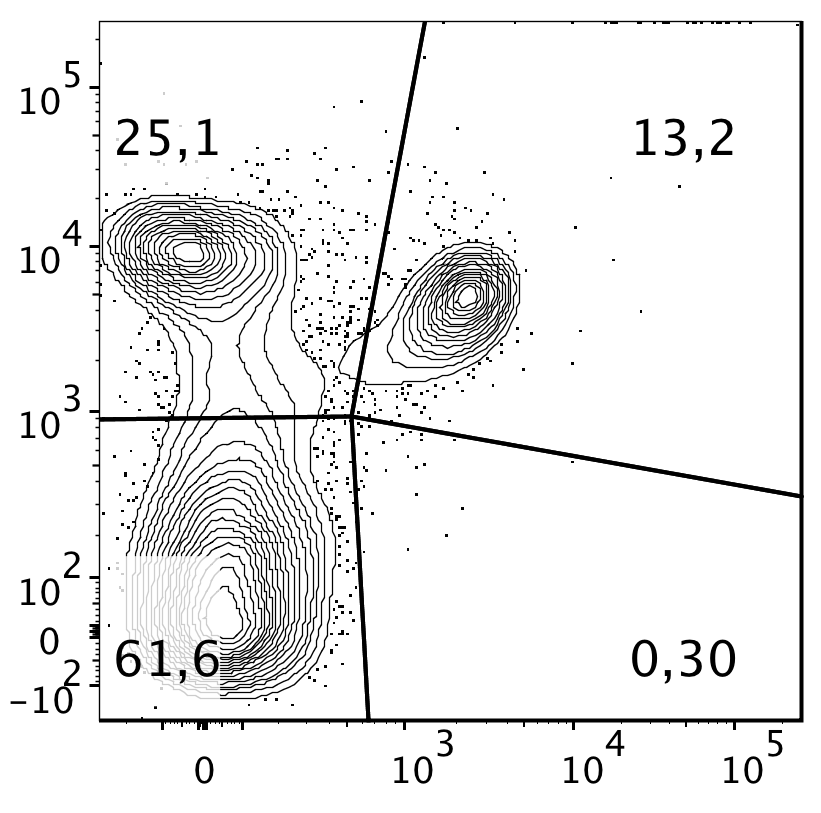

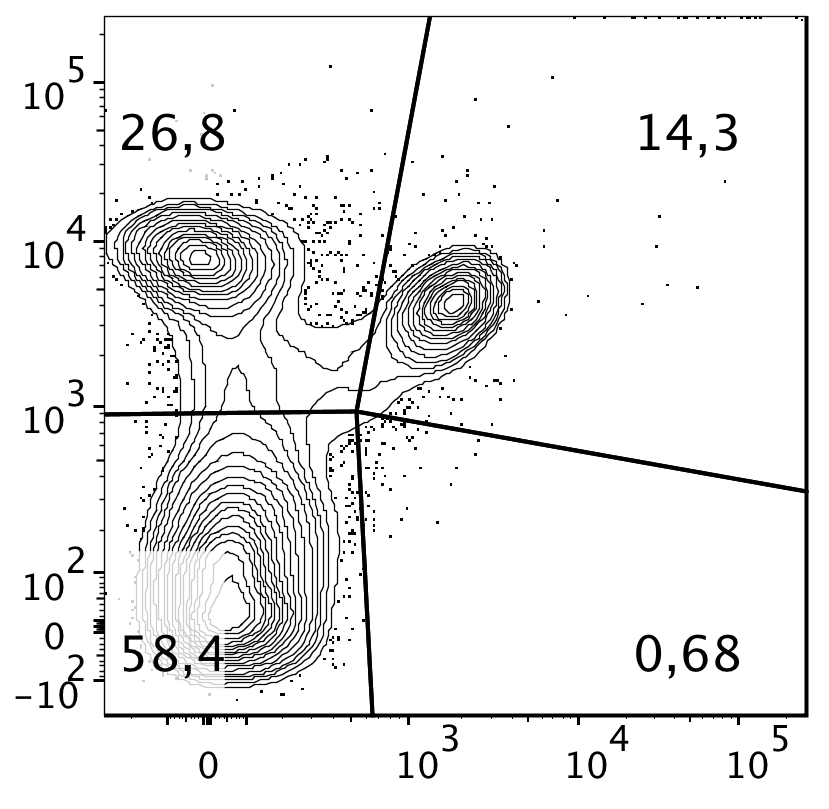

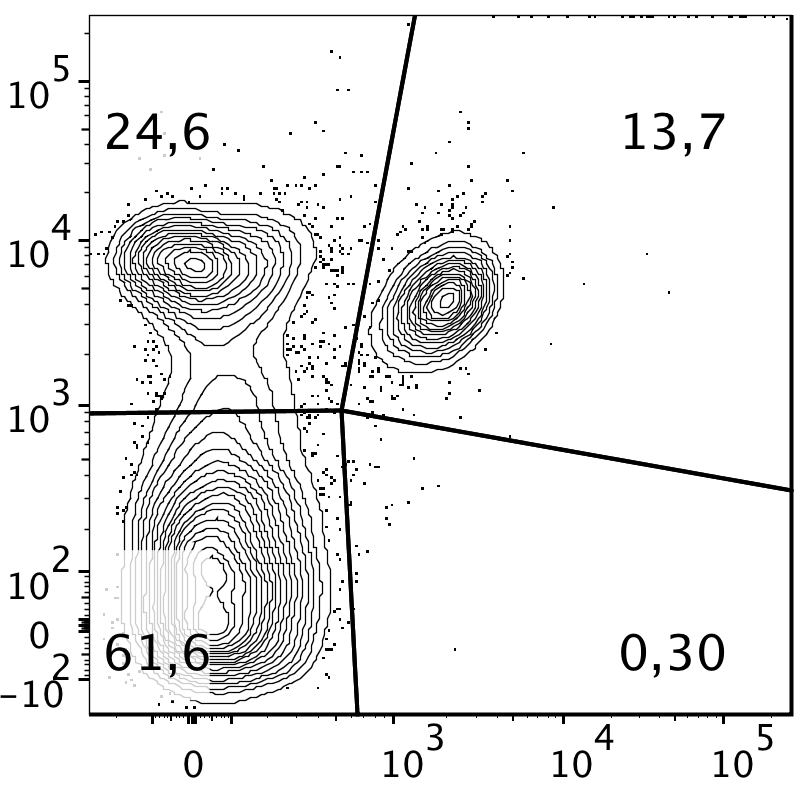


CD3

CD8


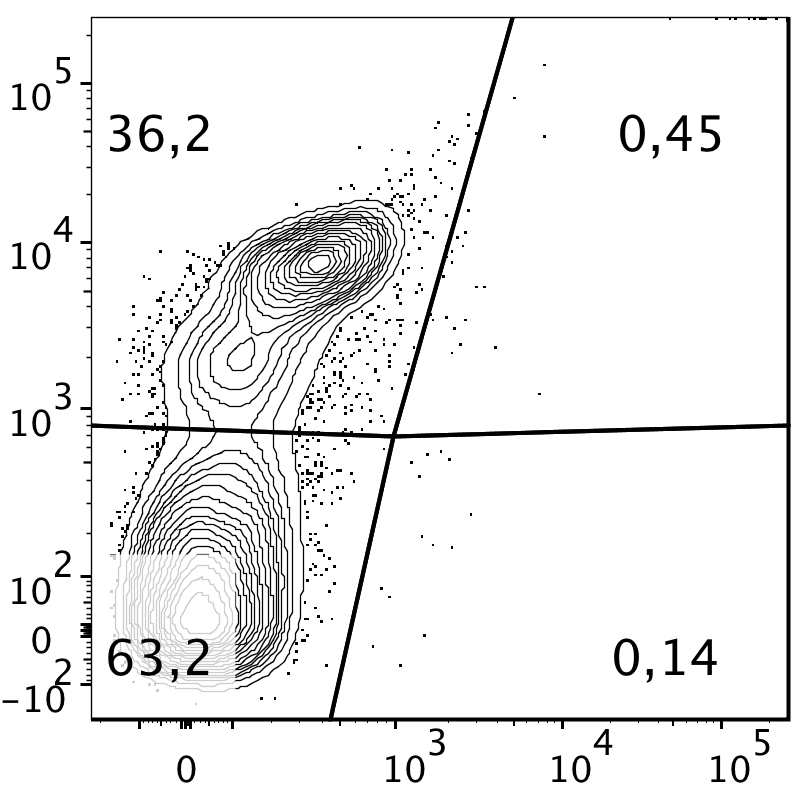

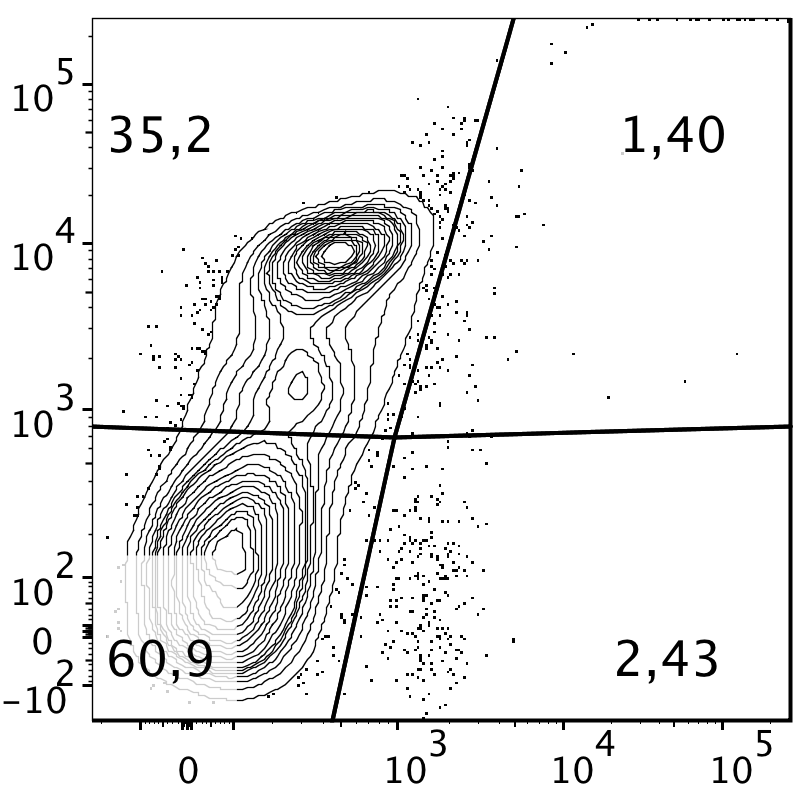

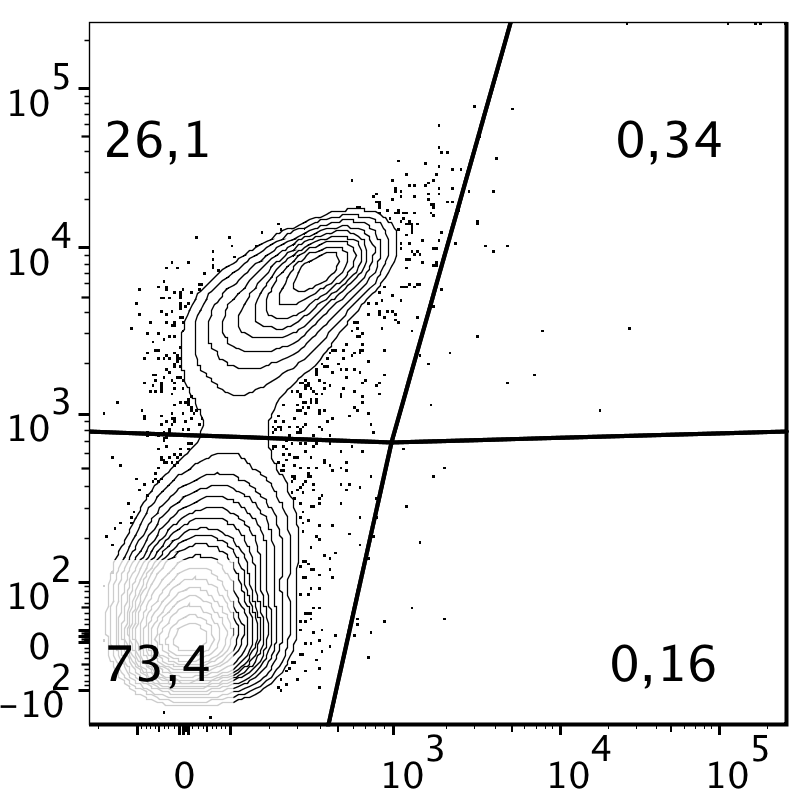

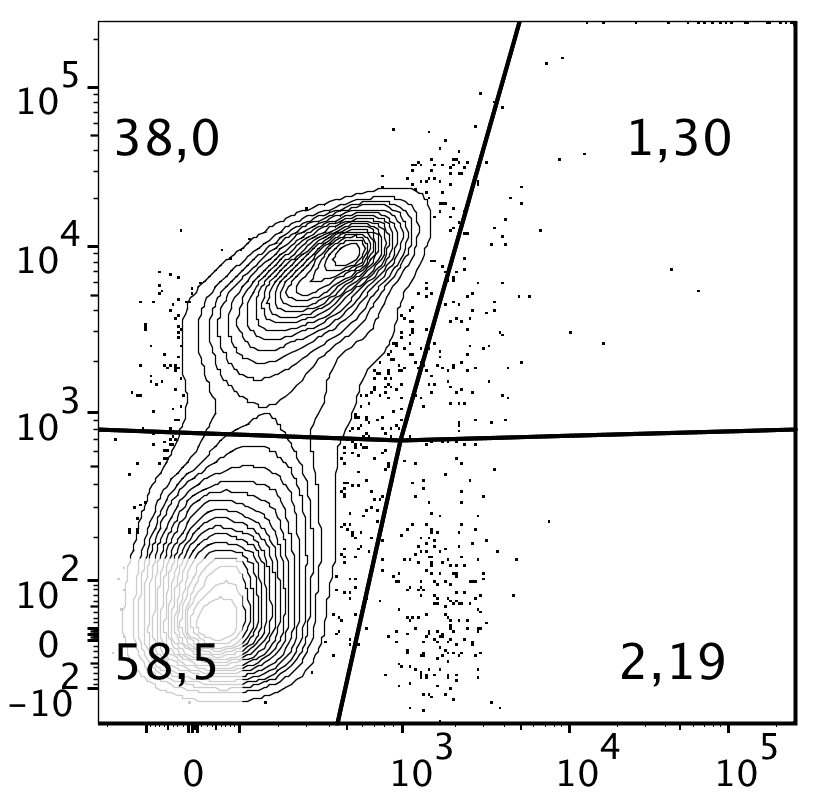

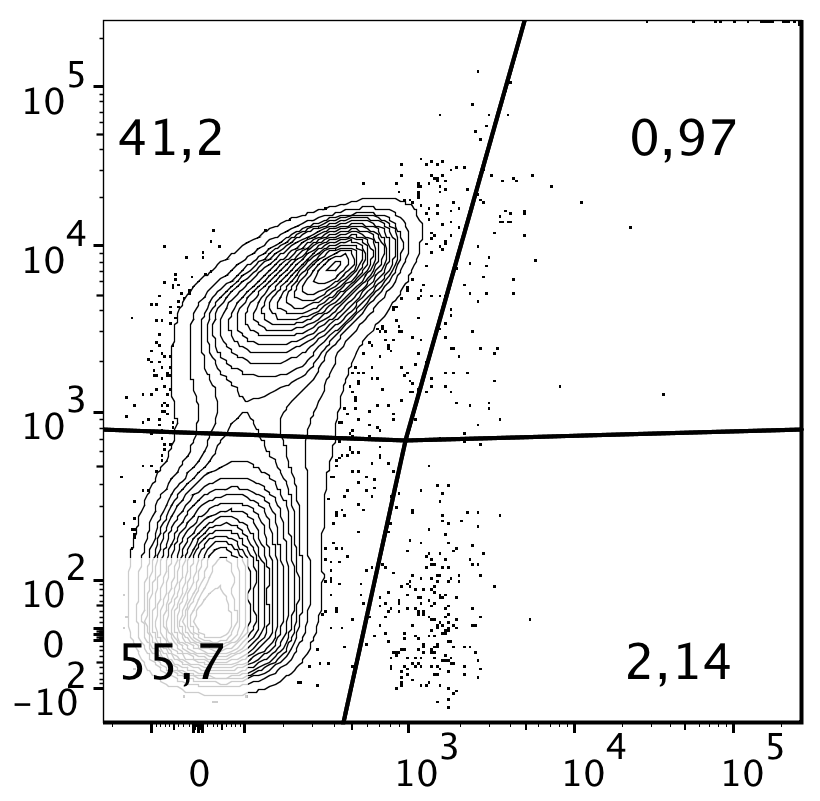

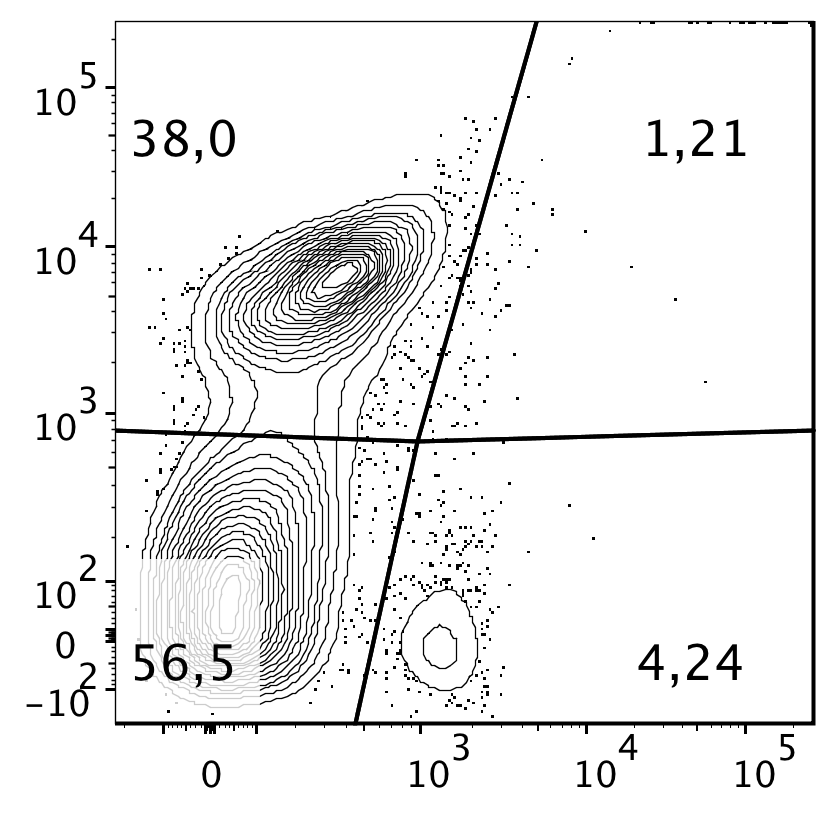


PBS

DCVacc/VSV-GP

isotype

aCD8

aNK

aNK/aCD8

CD3

NK1.1

A

B

w/o

**Suppl. Figure 7. CD8 T cell and NK cell depletion *in vivo* with anti-CD8 and anti-NK1.1 mAbs.** B16-OVA melanoma in C57BL/6 mice were injected i.p. with anti-NK1.1, anti-CD8 or isotype controls IgG2a and IgG2b in 100 µl PBS on day 8, 10, 14, 17 and 21 post tumor transplantation. Treatment with DCVacc/VSV-GP was started 3 days after the first injection of the depleting antibodies and repeated twice in 7 days interval. The percentages of CD8 T cells and NK cells in blood were determined by flow cytometry 3 days after the 3^rd^ DCVacc/VSV-GP treatment. (**A**) FACS dot plots depicting CD3 positive (y-axis) and CD8 positive (x-axis) cells (upper row) or CD3 negative (y-axis) and NK1.1 positive cells (lower row) show representative data from the different treatment groups. (**B**) Summary graphs of n= 1-6 mice per group from one experiment is shown for the percentage of CD3+CD8+ T cells and CD3-NK1.1+ cells.

**
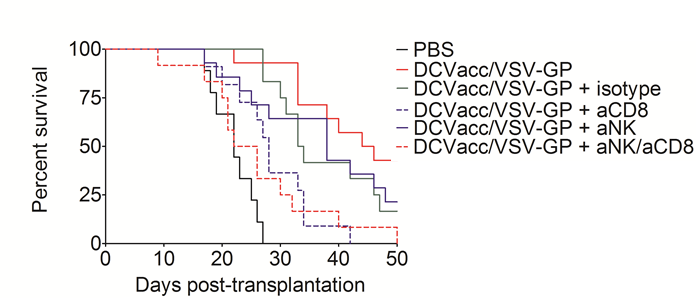
**

**Suppl. Figure 8**. **Depletion of CD8 T cells but not NK cells abrogated the therapeutic effect of DCVacc/VSV-GP.** B16-OVA melanoma in C57BL/6 mice were treated i.t./p.t. on days 11, 18 and 25 post transplantation with PBS (control) or DCVacc/VSV-GP (2×10^5^ OVA-loaded CpG-matured bmDCs/6×10^7^ PFU VSV-GP). CD8 T cells, NK cells or both together were depleted in DCVacc/VSV-GP treated animals by i.p. injection of specific antibodies or isotype controls at day 8, 10, 14, 17 and 21. Overall survival of mice is shown. Data were analyzed by Mantel-Cox test. The DCVacc/VSV-GP treated group showed significantly longer survival to PBS group (p<0.0001), CD8 T cell depleted (p ≤ 0.05) and NK/CD8 T cell depleted group (p ≤ 0.05). Results from 2 experiments with N=5-7 mice per group/experiment are represented.


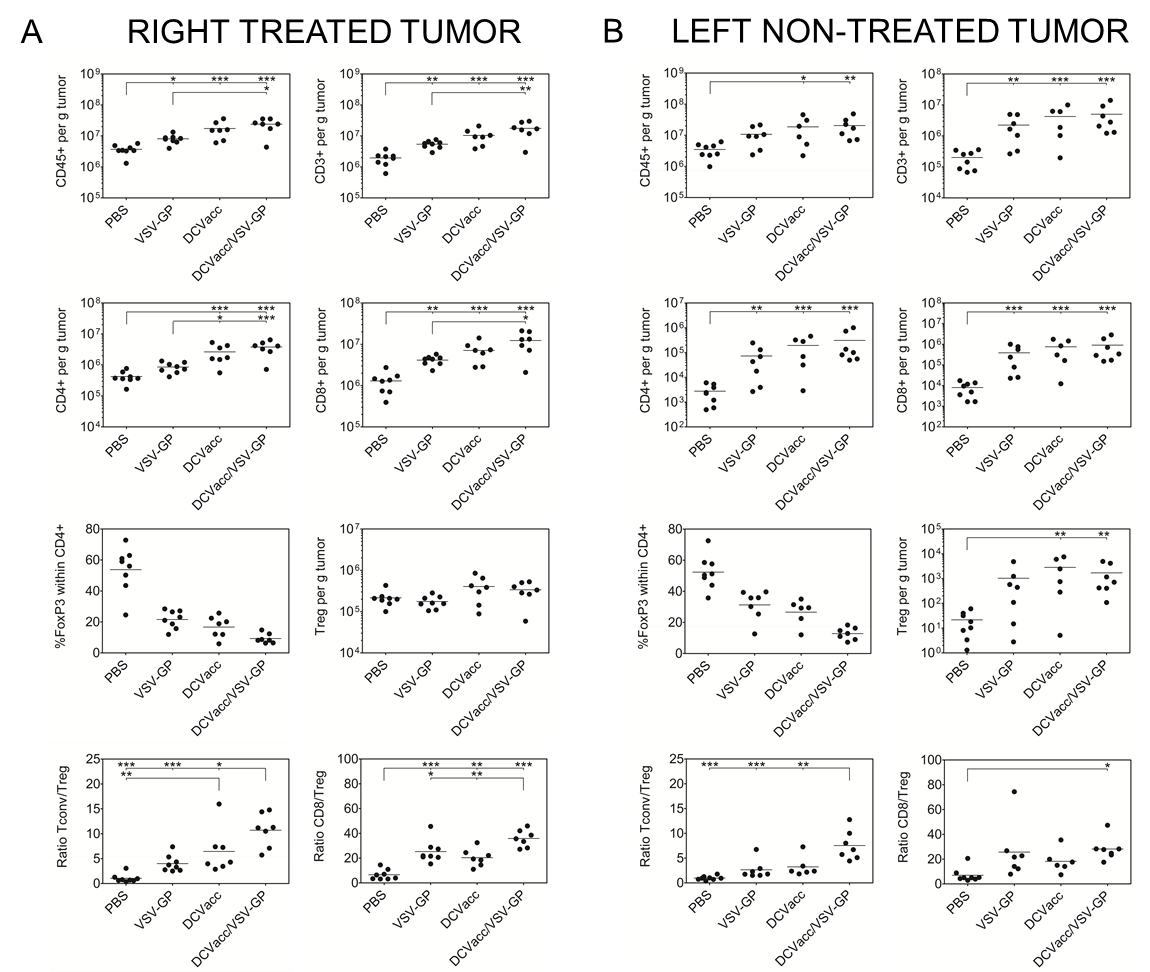


**Suppl. Figure 9. DCVacc/VSV-GP combination treatment increased lymphocytes infiltration and Tconv/Treg and CD8+/Treg ratios in treated and non-treated contralateral tumors.** B16-OVA melanoma cells were injected s.c.in the right flank and 3 days later in the left flank in C57BL/6 mice. Right flank tumors were treated i.t./p.t. on day 10 post transplantation with PBS (control), VSV-GP (6×10^7^ PFU), 2×10^5^ OVA-loaded CpG-activated bmDCs (DCVacc) or DCVacc/VSV-GP (2×10^5^ bmDCs/6×10^7^ PFU VSV-GP). Left flank tumors remained untreated. 7 days after the treatment, tumor cells were isolated and TILs were analyzed by FACS. Total cell numbers per g tumor were counted for CD45 positive immune cells, CD3 T cells, CD4 T cells, CD8 T cells and CD4/FoxP3 double positive Tregs as well as ratios Tconv/Treg and CD8/Treg were calculated in treated (**A**) and non-treated (**B**) tumors. Data represent results from one experiment. Data were analyzed by ANOVA followed by Tukey‘s multiple comparisons test (p ≤ 0.05 (*), p ≤ 0.01 (**), p<0.001 (***), p<0.0001 (****)).


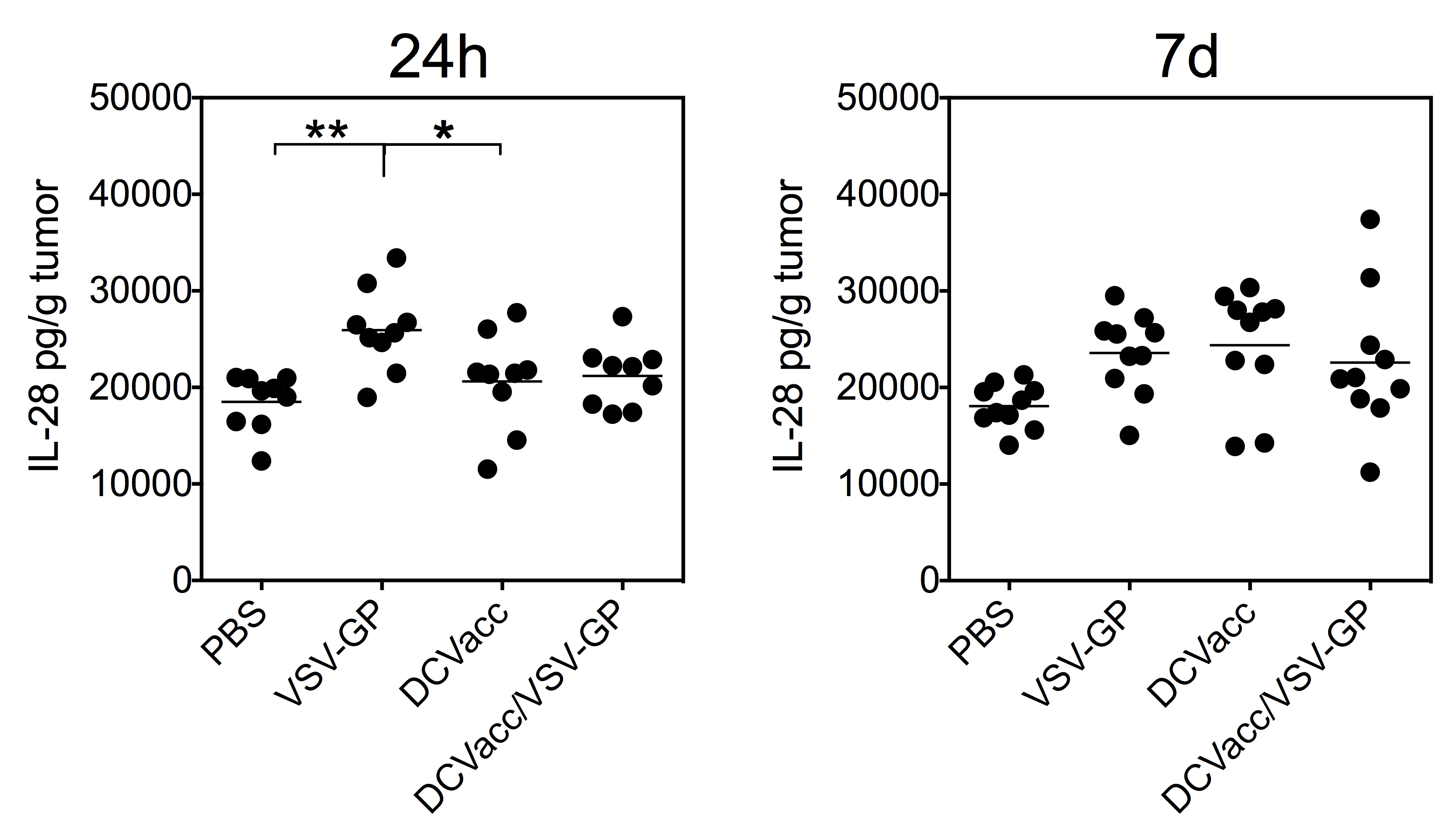


**Suppl. Figure 10. IL-28 in tumors after VSV-GP and DCVacc single and combination treatments.** B16-OVA melanoma in C57BL/6 mice were treated i.t./p.t. on day 11 post transplantation with PBS (control), VSV-GP (6×10^7^ PFU), DCVacc (2×10^5^ OVA-loaded CpG-matured bmDCs) or DCVacc/VSV-GP. IL-28 was determined in tumor lysates 24 hours (24h) and 7 days (7d) post treatments by ELISA. Data represent results of 2 independent experiments. Data were analyzed by ANOVA followed by Tukey‘s multiple comparisons test (p ≤ 0.05 (*), p ≤ 0.01 (**), p<0.001 (***), p<0.0001 (****)).
